# Analysis of Consumer Purchasing Behavior in Pet Food Industry: Marketing Mix Strategies Across Dog and Cat Owners

**DOI:** 10.3390/ani16213462

**Published:** 2026-11-02

**Authors:** Jona Danaj, Dimitra V. Liagka, Konstantinos Tousis, Artemis Konstantinou, Konstantinos Marinakos, Dimitrios Kantas, Elisavet Giamouri, Vasiliki Spyrou, Natalia G. C. Vasileiou, Maria Spilioti

**Affiliations:** 1 Faculty of Animal Science, University of Thessaly, 41110 Larissa, Greecespimaria1@gmail.com (M.S.); 2 Faculty of Crop Science, Agricultural University of Athens, 11855 Athens, Greece; 3 Independent Researcher, 11855 Athens, Greece; 4 Department of Tourism Management, University of West Attica, 12243 Athens, Greece

**Keywords:** pet food marketing, pet humanization, consumer involvement, MANCOVA, food labeling, premium pet food, information processing, distribution channels, market segmentation, trust in scientific authority

## Abstract

The global pet food market is influenced by the growing number of cats and dogs, the humanization of pets and the changing expectations of dog and cat owners. This study examines the role of marketing strategies in the dog and cat food market, focusing on the attitudes of Greek survey respondents, recognition of specific food categories, and factors associated with purchasing behavior. Data were collected through an online survey of 526 pet owners and guardians (*n* = 249 dog owners and *n* = 277 cat owners). The behavior toward pet foods containing selected premium ingredients was significantly linked to pet type (*p* = 0.021), the importance attributed to food ingredients (*p* < 0.001), veterinarian recommendations (*p* = 0.009), and the availability of scientifically substantiated information (*p* < 0.001). Among all factors, the importance of ingredients emerged as the factor displaying the strongest statistical association with consumer behavior (*p* < 0.001). Based on these findings, the study proposes a targeted marketing mix addressing product transparency, pricing, distribution, and promotion. The results contribute to the literature on pet humanization and provide practical guidance for pet food businesses, while highlighting the need for clearer regulatory definitions and scientific substantiation of marketing claims.

## Simple Summary

Globally, dogs and cats are an important part of many households, and owners are increasingly concerned about the quality and suitability of the food they provide to their pets. As a result, pet food companies need to better understand what influences owners when choosing different types of pet food. This study examined how the surveyed dog and cat owners in Greece make these purchasing decisions and what information they considered important. An online survey of 526 pet owners was conducted, including 249 dog owners and 277 cat owners. The results showed that owners’ choices of premium pet foods were influenced by the type of pet they owned, the importance they placed on food ingredients, advice from veterinarians, and the availability of reliable scientific information. Among these factors, the ingredients used in pet food had the greatest influence on purchasing decisions. The findings also showed that dog and cat owners may rely on different types of information depending on the food they are purchasing. These results can help pet food companies develop clearer and more informative products and communication strategies.

## 1. Introduction

Nowadays, people often develop strong bonds with their pets. These animals provide emotional support and companionship to their owners [1,2]. They also participate in daily family activities, such as group walks or play, contributing to the formation of the social fabric of the family. As a result, they are often treated as core members or best friends [3,4,5]. For these reasons, more and more pet guardians invest significant resources and time in caring for their pets, as they would in raising their own children [6,7]. The humanization of pets makes their owners particularly responsible in terms of providing adequate medical care and quality nutrition [8], because they want to enjoy their companionship for as many years as possible [9,10].

To achieve this longevity, proper nutrition for pets can be considered a preventive measure against various diseases [11,12]. Critical nutrients such as omega-3 fatty acids, various antioxidants, probiotics and prebiotics may provide anti-inflammatory and immunomodulatory effects [12,13,14]. At critical stages of a pet’s life, such as during aging, when various diseases such as obesity, osteoarthritis and cognitive dysfunction coexist, adapting the diet to their specific needs can prolong and improve their quality of life [15,16,17].

Pet food, which includes the diets developed for them, has evolved over time and aims to provide balanced nutrition for every phase of a pet’s life, using quality and safe nutrients [18,19]. Some of the consumer trends that prevail in human nutrition have also penetrated the feeding of companion animals, leading to the development of a wide variety of special food product lines such as those with organic ingredients, grain-free options, or formulas with meat of high biological value, low in fat and cholesterol, such as novel alternative protein sources (e.g., rabbit) [20,21,22]. These specialized product lines have been developed to meet changing consumer preferences and respond to the marketing strategies of pet food manufacturers, yet these products are significantly linked to the nutritional management of the health and well-being of companion animals [23]. The pet food market is saturated with various marketing claims regarding health safety or life stages, directly affecting actual nutrient intake and the suitability of the diet provided to the animals [24]. Misleading claims and confusing packaging labels can lead to inappropriate dietary choices, potentially exacerbating various clinical conditions. Therefore, evaluating how pet food marketing influences purchasing decision-makers is essential for understanding the broader context of animal welfare [25].

Globally, the size of the animal feed market is estimated to be between US$130 billion and US$160 billion [26]. This sector is showing a steady upward trend and is expected to exceed US$200 billion by 2035, recording a compound annual growth rate (CAGR) of approximately 6.5% [27]. North America holds the largest share of the global market, accounting for 41%. It is followed by Europe, while the Asia-Oceania region is showing the fastest growth rate [26]. Following this global expansion, the Greek pet food market has shown strong upward momentum, with total sales reaching €61 million in 2023 and projected to reach €66 million by 2028 [28]. Pet food imports in Greece recorded a remarkable compound annual growth rate (CAGR) of 10.81% between 2020 and 2024 [29].

Dogs and cats are the most popular pets. Globally, there are an estimated 840 million dogs and cats living in homes. This number corresponds to approximately 56% of all pet owners in the global population. Worldwide, there are approximately 471 million pet dogs, while there are 373 million cats. In the United States, dogs dominate the numbers, with 65 million owners, while cats follow with 49 million owners. In Europe, almost half of all households have some type of pet. Specifically, cats appear to be slightly more popular (129 million) than dogs (106 million). Russia has the highest number of cat owners, while Asian countries are experiencing the fastest growth in cat ownership, due to limited space in urban homes [30,31,32]. Within this context, and reflecting the absolute dominance of these two species over other pets in Greece, cats, due to the increase in urban living, are showing a steady population increase (reaching 606,000 in 2023) in contrast to the decreasing trend of dogs, although the latter remain more numerous in absolute numbers (655,000) [33].

In order to claim a valuable share in this dynamic dog and cat market, pet food companies need to develop specialized marketing strategies to meet the specific needs of pet owners [21,34,35]. The marketing mix can function as a basic strategic framework for dog and cat food companies to achieve their commercial goals [36,37]. The marketing mix consists of four basic elements, referred to as the 4Ps [38]. The first P concerns the product and is related to the characteristics of the food, such as quality and the provision of functional benefits, which could be influenced by the use of premium ingredients or the avoidance of grain, reflecting broader value chain premiumization frameworks [39]. The overall corporate identity, as well as the packaging of the food, are among the issues to be investigated for the product element [38,40,41,42]. The second P is related to price, i.e., the amount of money the customer is required to pay to obtain the specific food. The quality-price ratio can influence the consumer choices of pet owners, who are often willing to pay high prices in an effort to ensure the health of their animals through the effect of nutrition [37,43]. The third P refers to place, i.e., the channels from which the customer can purchase the pet food. In recent years, e-commerce and specialized pet shops have been added to the existing traditional sales channels of pet food, such as supermarkets and veterinary clinics [44,45]. The last P is related to promotion, including all communication actions, so that the target public is informed about the product being marketed and convinced to complete the purchase. The recent rapid rise of social media, influencers and content creators, seems to be affecting the classic way of advertising [46,47].

This research focuses exclusively on dog and cat owners in Greece, deliberately excluding owners of other pet species, since the animals under study constitute the largest part of the pet population, exerting a significant impact on the pet food industry [34,48]. The ever-increasing prevalence of high-quality pet foods for these species in the global market highlights the economic impact on this sector [40]. A critical synthesis of contemporary literature reveals that, while significant research attention has been devoted to the clinical biochemistry of pet nutrition and its primary economic implications, empirical research remains limited regarding how dimensions of strategic marketing interact with the multifaceted behavioral patterns of pet owners [25,34,41]. The strategic marketing mix (the 4Ps) does not constitute a causal framework; rather, it serves as a structured managerial classification system for the systematic categorization of external stimuli. Grounded in Consumer Involvement Theory [49] and the foundational constructs of the Theory of Reasoned Action (TRA) [50], these marketing stimuli—comprising product attributes, pricing cues, distribution channels, and promotional media—function as informational inputs that are processed through the consumer’s cognitive environmental filters. Specifically, the study examines how sociodemographic data (e.g., net household income) and pet ownership characteristics—differentiating between types of pets (e.g., dogs kept for guarding purposes versus cats)—relate to the formation of dynamic informational trust and behavioral purchase intentions. By identifying these cross-sectional interactions, this study addresses an under-researched topic in contemporary literature, exploring how various retail stimuli shape consumer decision-making pathways within the high-end (premium) pet care sector [25,34].The central research question examines customer behavior in the pet food market and specifically investigates how trust factors, exposure to advertising, and product characteristics shape purchase intentions across different consumer groups and pet species. In this context, the opportunities and challenges faced by pet food production and marketing businesses are documented, providing comprehensive marketing strategies for a better understanding of modern pet care.

The article uses primary and secondary data collected through a literature review and field research. The manuscript consists of five sections: introduction, materials and methods, results, discussion and conclusions.

## 2. Materials and Methods

### 2.1. Aim and Objectives

The purpose of this research is to evaluate consumer purchase intentions and media engagement patterns within the pet food market, focusing on distinct segments of dog and cat owners in Greece. Utilizing the strategic framework of the marketing mix (4Ps), the study systematically examines how external commercial stimuli interact with key household characteristics to shape the procurement pathways of pet owners.

The specific objectives of the study are:

1. To assess exposure frequencies to advertising and consumer preferences regarding specific visual marketing communication channels (Promotion Strategy).
2. To analyze product selection criteria and retail channel preferences across various household income brackets (Price and Place Strategies).
3. To identify the multivariate predictive power of multi-channel trust dimensions—such as veterinary guidance, ingredient evaluation, and scientific substantiation by the brand—on final purchase intentions for premium and specialized formulations (Product Strategy).

### 2.2. Research Questions and Research Hypotheses

In the light of the literature review, the following research questions and hypotheses were formulated:

Research Questions

The study examines the following fundamental research questions:

1. How do the frequencies of exposure to advertisements and preferences regarding the format of visual media (videos, photographs, content creators) vary based on the owner’s household net income and the type of pet?
2. To what extent do product selection criteria (brand equity) and food purchasing channels (pet stores, online shops, veterinary clinics) differ across various socioeconomic strata and pet types?
3. How do pet species and specific dimensions of trust in information—such as the evaluation of ingredients, veterinary recommendations, and scientific substantiation—predict consumer purchase intentions regarding premium pet food products and specialized functional formulations?

Research Hypothesis
Hypothesis 1

**H1a.**  *The frequency of exposure to advertisements and owners’ preferences regarding specific visual content channels (specifically photographic content, videos, and posts by content creators) are similar, regardless of the owner’s household net monthly income and the type of pet (dog vs. cat).*

**H1b.**  *The frequency of exposure to advertisements and owners’ preferences regarding specific visual content channels (specifically photographic content, videos, and posts by content creators) differ significantly based on the owner’s household net monthly income and the type of pet (dog vs. cat).*

Hypothesis 2

**H2a.**  *Key product selection criteria (specifically brand importance and packaging evaluation) and distribution channels (specifically specialized pet stores, e-commerce, and veterinary clinics) are the same across all consumer profiles, regardless of the owner’s net monthly household income and the type of pet (dog versus cat).*

**H2b.**  *Key product selection criteria (specifically brand importance and packaging evaluation) and distribution channels (specifically specialized pet stores, e-commerce, and veterinary clinics) differ significantly based on the owner’s net monthly household income and the type of pet (dog versus cat).*

Hypothesis 3

**H3a.**  *The type of pet (dog versus cat) and the key dimensions of trust in product information (specifically: the importance of ingredients, professional recommendations from veterinarians, and the brand’s scientific substantiation) do not significantly predict purchase intentions for premium and specialized pet foods.*

**H3b.**  *The type of pet (dog versus cat) and the key dimensions of trust in product information (specifically: the importance of ingredients, professional recommendations from veterinarians, and the brand’s scientific substantiation) significantly predict purchase intentions for premium and specialized pet foods, with dog and cat owners exhibiting distinct response profiles.*

### 2.3. Sample

**Figure 1:**
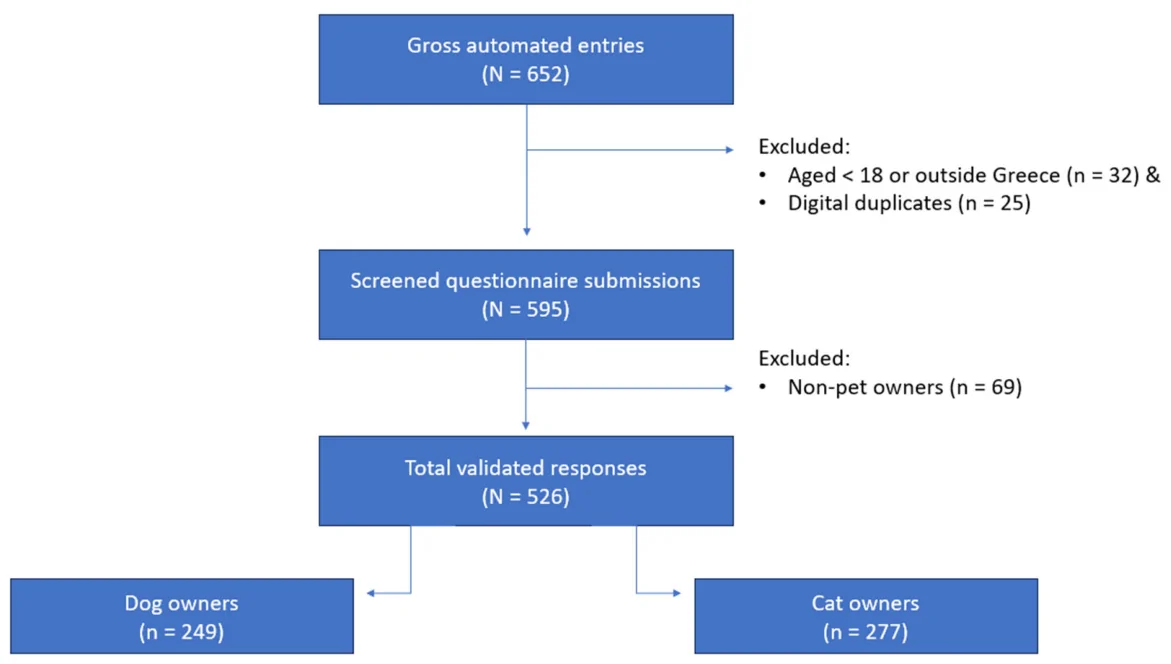
Participant flow diagram and multi-stage data-cleaning filtration layout.

Primary data collection took place between January and February 2026, recording a total of *N* = 652 initial accesses to the digital survey. Following a multi-stage screening and filtering process, 126 entries were excluded (specifically: 32 entries from individuals under the age of 18 or residing outside Greece, 25 digital duplicates, and 69 individuals who are not pet owners), resulting in a final, validated empirical sample of 526 responses (*n* = 526), which was collected via non-probability “snowball” sampling (see Figure 1). The questionnaire was distributed primarily via social media platforms (such as Facebook and Instagram), leveraging popular Greek online communities for pet owners as well as professional networking pages for veterinarians. The sample consisted exclusively of key decision-makers (pet owners/guardians), divided into two distinct categories based on pet ownership: dog owners (*n* = 249) and cat owners (*n* = 277). From a socio-economic perspective, the sample reflects a distribution predominantly within the middle class; however, the extreme income strata are smaller—specifically the high-income group (>€2500: *n* = 11 for dogs, *n* = 8 for cats) and the lower-income group (≤€500: *n* = 14 for dogs, *n* = 27 for cats)—a factor that was taken into account when conducting inferential statistical tests using robust variance estimators. This specific target group was chosen because their dietary perceptions, behavioral orientations, and purchasing choices directly shape product development and marketing performance indicators in the pet food sector. Each participant could only complete the survey once. From a technical standpoint, duplicate submissions were identified and electronically blocked using automated timestamp logging and unique digital browser session identifiers embedded in the survey software. Eligibility criteria required participants to be dog or cat owners residing in Greece, aged 18 or older, and to bear primary or shared responsibility for procuring their pets’ food. To ensure strict sample independence and avoid analytical confounding, the study was conducted through two separate, dedicated survey instruments developed through separate digital links for dog and cat owners, respectively. Consequently, participants owning both types of animals were explicitly instructed at the beginning of the questionnaire to select and complete the section pertaining solely to the pet for which they held primary care responsibility. Furthermore, given that all key question sections on the digital survey platform were designated as mandatory fields, the completion rate of submitted questionnaires remained at 100%, ensuring the absence of missing data at the submission stage. As part of the assessment of sample adequacy and statistical power, a post-hoc statistical power analysis was conducted based on the current sample size (*N* = 526). For the multivariate predictive models (MANCOVA and multiple linear regression models)—assuming a highly conservative effect size in the small-to-medium range (f^2^ = 0.035), a significance level of α = 0.05, and up to four initial predictor variables—the empirical statistical power exceeded 0.95 (1 − β = 0.95). This demonstrates that the current sample is mathematically robust and sufficient for identifying stable associations (at a given point in time), without posing a risk of Type II errors. Furthermore, given that sampling was conducted online using the “snowball sampling” method—a non-probability approach based on open, dynamic links shared on social media—it is not possible to mathematically calculate an exact response rate, as the total number of individuals exposed to the survey (the denominator representing total reach) remains unknown. Nevertheless, monitoring of the process confirmed full structural completion (a 100% completion rate) for all digital submissions that were finalized.

### 2.4. Operationalization of Variables and Research Instrument Development

**Figure 2:**
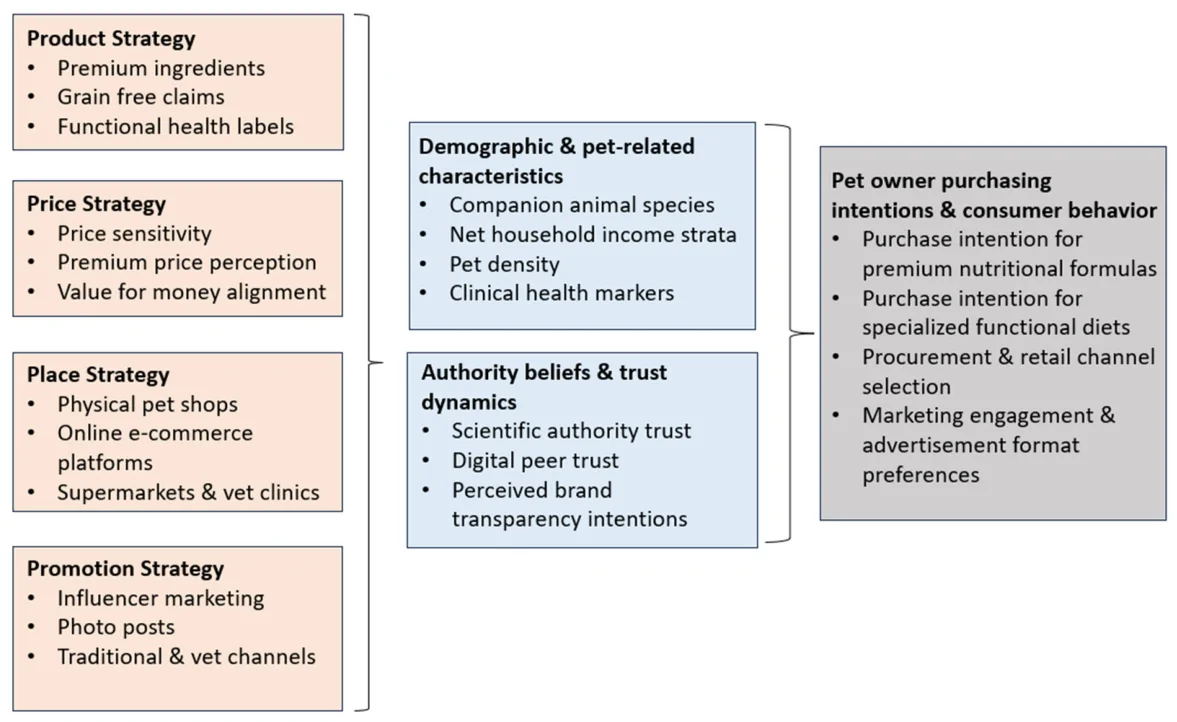
Conceptual framework aligning marketing mix (4P) strategies for pet food with consumer purchasing behavior and the attitudes of pet owners.

Figure 2 depicts the conceptual framework that focuses on the strategic alignment of pet food marketing mix strategies (4Ps) with consumer purchasing behavior and owner attitudes. A process structure of this type serves as a critical methodological tool, visually representing the interconnection of marketing stimuli, environmental filters, and behavioral outcomes, thereby facilitating a better understanding of complex consumer decision-making. This visualization was developed using Microsoft PowerPoint, Microsoft 365 Edition (Microsoft Corp., Redmond, WA, USA), to effectively bridge the literature review and operationalize the key dimensions of the study. The analytical structure of the framework is divided into main sequential pillars, which are operationalized through the specific elements of the marketing mix: Product Strategy captures inherent attribute evaluations, including high-quality ingredient claims, grain-free formulation labels, and functional health claims that reflect the contemporary phenomenon of pet humanization. Price Strategy assesses consumer pricing orientations and sensitivities, evaluating perceptions of high price-quality and value-price alignments. Place Strategy reflects distribution preferences across all channels, covering traditional specialty retail (pet stores), digital e-commerce platforms, and professional referral points such as veterinary clinics or supermarkets. The Promotion Strategy integrates marketing communications, focusing on influencer marketing, content creation engagement, and professional and traditional channels. Through the incorporation of structural brackets, the model demonstrates that these unified marketing stimuli are systematically filtered through two central contextual dimensions: demographics and pet-related characteristics (such as pet species, income strata, and clinical health indicators) and beliefs and trust dynamics (balancing scientific veterinary trust versus digital peer trust). By mapping these dimensions from initial strategic inputs to pet owners’ ultimate purchase intentions and consumer behaviors, we ensured that subsequent questionnaire items fully captured all empirical aspects of the proposed consumer marketing framework.

In this context, a structured questionnaire consisting of 32 questions for dog owners was used as a research tool (the version for cat owners contained 31 questions, as Question 6—regarding physical confinement, i.e., free-roaming versus being chained or kept in a cage—was omitted, given that it is not applicable to cats; see Supplementary File S1). The questionnaire was divided into three parts. To directly examine the study’s core hypotheses, a specific set of 35 targeted questions was selected from these sections for further analysis (this specific number arises from the fact that several sections of the questionnaire were structured as tables with Likert scales—for instance, the question concerning claims on product labels included 10 individual items, which were evaluated independently as separate variables during the analysis in SPSS), as systematically presented in Table 1. 

**Table 1:** Methodological mapping of survey questionnaire items, variables and statistical frameworks.

Survey Section & Specific Questions	Extracted Sub-Items/Targeted Inferential Variables	Operationalized Measurement Scale	Associated Research Hypothesis	Inferential Statistical Analysis Executed
Part 1: general pet & owner data Questions: Q2 & Q30	Baseline demographic context (analyzed as categorical strata and independent sample filters)	Nominal/categorical ordinal strata categorical groups	Covariates/independent filters	Descriptive frequencies baseline demographic matrices
Part 2: Advertising & promotion Questions: Q19 & Q23	Q23: Media format preferences (5 items) Q19: Medium exposure frequencies (5 items)	5-point Likert scale 5-point Likert scale	Hypothesis 1 (H1a/H1b) (Promotion Strategy)	One-way ANOVA, Welch’s robust test, Kruskal–Wallis H test, and Bonferroni post-hoc tests
Part 3: Consumption values & trust Questions: Q15, Q16, Q18, Q21, Q25	Q15: Label claim attributes (10 items) Q18: Food selection criteria (7 items) Q16: Procurement channels (4 items) Q21 & Q25: Core information trust metrics (4 targeted items evaluating veterinary recommendations and scientific substantiation)	5-point Likert scale 5-point Likert scale 5-point Likert scale 5-point Likert scale 5-point Likert scale	Hypothesis 2 H2a/H2b (Price & Place) Hypothesis 3 (H0/H1) H3a/H3b (Product strategy)	-One-way ANOVA & Robust Welch -Multivariate Analysis of Covariance (MANCOVA) -Multiple Linear Regression, GLM (Robust SE) & Ordinal Logistic Regression -Pearson & Spearman Bivariate Correlations

Note: The numbering of the questions in Table 1 and throughout the text follows the standardized 31-question structure used for cat owners (in the version for dog owners, which comprises 32 questions, the inclusion of Question 6—regarding animal confinement, which is species-specific—shifts the subsequent questions, from the 6th to the 31st, to positions 7 through 32; see Supplementary File S1). The sections of the questionnaire omitted from the table illustrating the inferential relationships (specifically questions Q1, Q3, Q4, Q5, Q6, Q7, Q8, Q9, Q10, Q11, Q12, Q13, Q14, Q17, Q20, Q22, Q24, Q26, Q27, Q28, Q29, and Q31, according to the standardized numbering, as well as the question concerning the confinement of dogs) were used exclusively for exploratory and descriptive statistical analysis—specifically to determine baseline frequencies, percentage distributions, and qualitative filters. Consequently, they served as fundamental points of reference—both in the broader demographic profile table (Table 2) and in the sections presenting the descriptive results and qualitative text analysis—rather than as variables with multiple predictive roles in subsequent multivariate analytical models.

**Table 2:** Demographic and basic characteristics of pet owner survey participants.

Variable	Categories (%) Dog Owners (*n* = 249)	Categories (%) Cat Owners (*n* = 277)
Gender	Male (11.6%)/Female (88.4%)	Male (6.9%)/Female (93.1%)
Age	18–24 (12.4%), 25–44 (52.2%), 45–64 (33.4%), 65 years and over (2.0%)	18–24 (10.2%), 25–44 (52.9%), 45–64 (34.9%), 65 years and over (2.0%)
Household income	≤€500 (6.4%), €501–1000 (44.7%), €1001–2500 (43.8%), over €2500 (5%)	≤€500 (11.2%), €501–1000 (40.7%), €1001–2500 (44.8%), over €2500 (3.3%)
Pet density	1 pet (72.4%), 2–3 pets (24.7%), >3 pets (2.9%)	1 pet (37.4%), 2–3 pets (37.4%), >3 pets (25.2%)
Breed type	Mixed breed (61.8%), Purebred (28.7%), Both (9.5%)	Mixed breed (88.1%), Purebred (5.1%), Both (6.8%)
Sterilization status	Neutered/Sterilized (69.7%), Intact (30.3%)	Neutered/Sterilized (93.5%), Intact (6.5%)
Living environment	Indoors (84.0%), Garden/Yard/Outdoors (16.0%)	Indoors (89.5%), Garden/Yard/Outdoors (10.5%)

(1) Sociodemographic characteristics and pet profile: 11 closed-ended questions for dogs (10 for cats, omitting the question regarding leashes/walking)—covering informed consent, number of pets, breed, neutering status, living environment, care during the owner’s absence, duration of ownership, the owner’s gender and age group, and household net income—along with two open-ended questions (concerning the pet’s age and general final comments), the data from which were analyzed both quantitatively and qualitatively. This section identified key consumer categories and care profiles among the participating dog and cat owners, serving as the primary demographic filters for the analysis of the marketing mix.
(2) Exposure to advertisements & Media interaction: 5 closed-ended questions and one open-ended question (regarding misleading marketing terms), which relate to Research Objective 1 (RO1). This section examines the frequency of exposure to marketing activities across various channels (television, digital media, social media, pet stores, and print media), attitudes toward the regulation of advertising, and the visual and thematic elements that capture consumer attention when processing advertisements. It focuses specifically on the “promotion” pillar of the marketing mix, evaluating consumer preferences regarding the format of media content.
(3) Assessment of attitudes, trust dynamics, and purchasing behavior: This includes 13 closed-ended questions directly related to Research Objective 2 (RO2)—exploring the recognition of specific label claims, dietary habits, monthly expenditure, and qualitative attributes as strategic purchasing drivers—and Research Objective 3 (RO3)—identifying information sources, brand perceptions, and the balance between trust in veterinarians (a scientific approach) and the influence of digital content creators (influencers). This unit covers the pillars of product strategy (label claims and quality ingredients), pricing strategy (brand value and the price-quality relationship), and distribution strategy (selection of distribution and supply channels).

Although participants had the opportunity to express their opinions in the open-ended question regarding confusing or misleading terms, no such responses were received.

The questionnaire was developed in accordance with the three research hypotheses of the study and aimed to capture the attitudes, preferences and behaviors of pet owners from multiple perspectives.

Regarding Hypothesis 1, the questions examined the frequency of respondents’ exposure to pet food advertisements and their preferences regarding the type of advertising content, specifically evaluating variables related to the promotion element of the marketing mix, including individual exposure and preferences regarding video ads, photo ads, and posts by content creators. This section addressed the frequency of advertisements appearing in various media channels in the last month (5 items on a 5-point Likert scale: 1 = never, 2 = rarely, 3 = sometimes, 4 = often, 5 = always; covering television, internet, social media, pet stores and print media). It also assessed participants’ preferences regarding the specific type of advertising content that most caught their attention (5 items on a 5-point Likert scale: 1 = not at all, 2 = a little, 3 = moderately, 4 = a lot, 5 = very much; including videos, photos, pet posts, influencers, and content creators).

Hypothesis 2 was tested through questions that explored product selection criteria and purchase/distribution channels used for pet food. Respondents rated the extent to which specific label features would prompt them to purchase a product (10 items on a 5-point Likert scale: 1 = never, 2 = rarely, 3 = sometimes, 4 = often, 5 = always; focusing on variables that are functionally specific to pricing strategy (specifically, the importance of the brand as a factor justifying a higher price due to the quality guarantee) and place strategy (evaluating consumer purchasing intentions across specialized pet stores, e-commerce, and veterinary clinics). In addition, we measured the frequency of purchasing pet food from different distribution channels (4 items on a 5-point Likert scale: 1 = never, 2 = rarely, 3 = sometimes, 4 = often, 5 = always; assessing pet stores, supermarkets, veterinary clinics, and online stores). The reported budget allocation was further integrated into this framework through an assessment of monthly expenditure on pet food. This approach serves as an indicative measure of a household’s financial constraints—rather than functioning as an isolated psychometric construct of absolute price sensitivity—given that actual spending on pet nutrition is naturally correlated with the demographic profile, age, and health status of the animals.

Hypothesis 3 focused on the behavioral responses of pet owners to the product strategy dimension of the marketing mix—specifically, the evaluation of claims regarding superior-quality ingredients and specialized life-stage formulations—in relation to key information sources and characteristics that inspire scientific trust, thereby providing the fundamental metrics for the predictive multivariate models. This part of the questionnaire assessed the level of influence exerted by various sources during the main pet food selection process (6 items on a 5-point Likert scale: 1 = never, 2 = rarely, 3 = sometimes, 4 = often, 5 = always; e.g., veterinarian recommendation, advertisements, or animal scientist recommendation). It also quantified the overall level of trust in these information sources (5 questions on a 5-point Likert scale: 1 = not at all, 2 = a little, 3 = moderately, 4 = a lot, 5 = very much; e.g., veterinarians, advertisements, influencers, pet stores, and social media).

In addition to the hypothesis-based questions, the questionnaire included informational and demographic questions to capture the basic profile of both the pet and the owner. These questions asked about pet characteristics (number of pets, breed, age), neutering status (yes/no/don’t know), and duration of pet ownership, along with owner demographics such as gender, age group, and monthly net household income.

The strategic design and selection of the research instrument’s items followed a rigorous psychometric evaluation protocol, aimed at ensuring construct validity and structural reliability. The questionnaire items were derived and adapted from established measures within the fields of marketing and the pet retail sector, as described in contemporary literature [34,51]; they were initially drafted in English and subsequently translated into Greek. To ensure semantic equivalence, a standardized forward-and-backward translation process was conducted by independent bilingual researchers. Initial clarity and face validity were assessed through a formal pilot study, involving a convenience sample consisting of 15 pet owners and 3 veterinarians. Content validity was confirmed by a panel of three external subject-matter experts (two distinguished academics in the animal sciences and one specialist in animal nutrition), who evaluated the alignment of the items with the research objectives using a structured Content Validity Index (CVI). This process yielded an excellent score at the scale level (S-CVI/Ave = 0.94). To assess the construct validity of the extracted behavioral dimensions (label attributes, brand perceptions, and trust dynamics regarding information), an Exploratory Factor Analysis (EFA) was conducted separately for each group of pet owners, using Principal Component Analysis (PCA) with Varimax rotation. The Kaiser–Meyer–Olkin (KMO) measure of sampling adequacy was 0.820 for dog owners and 0.803 for cat owners, while Bartlett’s Test of Sphericity showed high statistical significance for both groups (dogs: χ^2^ = 1486.15, df = 210, *p* < 0.001; cats: χ^2^ = 1103.15, df = 210, *p* < 0.001), confirming the suitability of the data for factor reduction. The individual items of the questionnaire were successfully grouped into five stable and mathematically independent behavioral constructs, which account for 59.64% of the total variance in the sample of dog owners and 60.05% in the sample of cat owners. Furthermore, the reliability and internal consistency of the scale were confirmed via Cronbach’s alpha coefficients, which ranged from 0.74 to 0.86 for all multi-item indices, thereby satisfying rigorous psychometric criteria for their inclusion in subsequent models involving multiple predictor variables.

### 2.5. Statistical Analysis

Data analysis was performed using the statistical software IBM SPSS Statistics, version 31 (IBM Corp., Armonk, NY, USA), employing a combination of descriptive and inferential statistical techniques. In accordance with established psychometric and econometric practices in consumer research, data from 5-point Likert scales were analyzed within parametric frameworks (MANCOVA, one-way ANOVA, and multiple linear regression using the ordinary least squares [OLS] method), supported by the large sample size (*N* = 526) and explicit diagnostic checks of the models. Specifically, for the MANCOVA model, the homogeneity of the covariance matrices was assessed using Box’s M test (Box’s M = 426.11, F(275, 11,684.58) = 1.28, *p* = 0.001), while the equality of error variances for all 10 dependent variables was examined via Levene’s test. The latter confirmed homoscedasticity for six items (*p* > 0.05), while indicating heteroscedasticity for four items (“grain-free,” *p* = 0.001; “gluten-free,” *p* = 0.044; “no additives/preservatives,” *p* = 0.011; and “for a specific category of animals,” *p* = 0.021). Consequently, priority was given to the Pillai’s Trace (V) criterion—alongside Wilks’ Lambda (Λ)—due to its proven robustness against violations of the homogeneity of covariances. For the OLS regression models, linearity and homoscedasticity were verified through visual inspection of scatterplots of standardized residuals against standardized predicted values (*ZRESID versus *ZPRED). The normality of the residuals was assessed using histograms of the standardized residuals and normal probability plots, while the independence of errors was confirmed via the Durbin–Watson test (values of 1.777 and 1.731, which fall within the acceptable range of 1.5–2.5). The absence of multicollinearity was confirmed via Variance Inflation Factors (VIF < 1.27), tolerance statistics (tolerance > 0.78), and eigenvalue condition indices (maximum Condition Index = 15.65, a value well below the critical threshold of 30). To further ensure that treating ordinal items from a 5-point Likert scale as approximations of continuous variables did not introduce estimation bias, extensive non-parametric and sensitivity analyses were conducted—specifically, Generalized Linear Models (GLM) with robust standard errors (Huber–White/sandwich estimators), Ordinal Logistic Regression (proportional odds/PLUM models), Kruskal–Wallis H tests, and Spearman’s rank correlation coefficients (rho)—within the context of the respective analytical models. These sensitivity checks confirmed strong directional and statistical consistency across the analyses, while any minor discrepancies between the parametric estimates and those derived from ordinal or robust models are explicitly noted in the results.

To evaluate the pre-established research hypotheses through a structured analytical design, specific statistical frameworks were systematically applied to each element of the strategic marketing mix. Hypothesis 1 (the Promotion pillar) and Hypothesis 2 (the Price and Distribution/Place pillars) were evaluated using one-way analysis of variance (one-way ANOVA), in conjunction with Levene’s test for the homogeneity of variances. In cases where heteroscedasticity was detected (*p* < 0.05), Welch’s analysis of variance (ANOVA)—which follows an asymptotic F-distribution—was employed as the primary estimation method to account for unequal variances and unequal group sizes across income strata. Post-hoc pairwise comparisons were conducted using Bonferroni corrections, accompanied by 95% confidence intervals (95% CIs) and Cohen’s d effect sizes. Hypothesis 3 (the “Product” pillar) was evaluated via a sequential two-phase approach: first, a Multivariate Analysis of Covariance (MANCOVA) was performed—utilizing Type III sums of squares—on the vector of 10 items pertaining to claims found on product labels. Both the Wilks’ Lambda (Λ) statistic and Pillai’s Trace (V) were recorded—the latter serving as a robust multivariate statistical criterion, resilient to potential deviations from multivariate normality and homogeneity of covariances—while effect sizes were calculated based on partial eta-squared (η^2^p). Subsequently, two multiple linear regression models were estimated to isolate the directional predictive power of key dimensions of trust; these models present unstandardized coefficients (B) with their corresponding 95% confidence intervals, standardized coefficients (β), and diagnostic checks for collinearity. Finally, to rigorously address the issue of multiple comparisons across various families of statistical tests (namely, the 10 univariate tests following the MANCOVA, the overall ANOVA/Welch tests for each marketing pillar, and the bivariate correlation matrices), the Benjamini–Hochberg procedure was applied to control the False Discovery Rate (FDR) at q = 0.05. The *p*-values obtained after FDR correction (q-values) are presented alongside the original *p*-values (raw *p*-values). For the multivariate frameworks the dependent variables included consumer purchasing behavior regarding various label attributes, such as the absence of preservatives, high protein content, low fat content, the use of natural preservatives, premium ingredients, grain-free formulation, gluten-free formulation, absence of additives or artificial preservatives, hypoallergenic properties, and suitability for specific animal categories (e.g., puppies, neutered pets, and animals following weight-control programs). The independent variables were pet type and monthly income. Key characteristics of companion animals—including neutering status, pet density, and breed type—were treated as intrinsic, species-specific structural dimensions that naturally characterize the distinct market profiles of dog-owning versus cat-owning populations, rather than as isolated confounding factors. Covariates included criteria used for selecting the pet’s primary food, namely product price, ingredients, nutritional value, food brand, veterinarian recommendation, advertising, and packaging characteristics. Additional covariates included attitudes toward brand name attributes, such as brand history and recognition, brand loyalty, corporate social responsibility activities, transparency regarding production processes, knowledge of company employees, and the importance of scientifically documented information about products. Furthermore, trust in scientific authority was incorporated as a covariate, including trust in veterinarians, advertising, influencers, pet shops, and social media sources. Based on the MANCOVA results, food ingredients, veterinarian recommendations, and the presentation of scientifically supported information emerged as significant factors influencing consumer purchasing behavior across different label characteristics. These effects were further examined for selected premium ingredients and product labels referring to specific animal categories. Multiple linear regression analyses were conducted to identify factors predicting consumer purchasing behavior when pet food labels highlight premium-quality ingredients or emphasize suitability for specific categories of animals. For the multivariate predictive frameworks, a standard complete-case analysis—utilizing listwise deletion—was applied to address invalid or missing responses; additionally, a multi-stage data-cleaning process was implemented for the model variables. Specifically, in the MANCOVA model—which incorporated the household’s net monthly income (Q30), covariates (Q18, Q21, Q25), and 10 dependent variables related to product labels (Q15)—certain observations were excluded, primarily because respondents selected the option “I do not wish to answer” regarding household income (Q30), or because they provided invalid responses within the questionnaire matrices. Regarding the next two multiple linear regression models, listwise deletion—accounting for the active predictors (pet type, influence of ingredients from Q18, veterinary recommendation from Q18, and scientific substantiation from Q25) as well as the corresponding dependent variable from Q15—resulted in the exclusion of 57 observations for the model concerning higher-quality ingredients (final effective sample size *n* = 469, F(4, 464)) and 64 observations for the model concerning specialized biological diets (final effective sample size *n* = 462, F(4, 457)). This rigorous, multi-stage data-cleaning process ensures that the regression coefficients remain statistically robust and free from systematic demographic bias.

Regarding the operational definition of the outcome measures in both regression frameworks, the dependent variables were derived directly from the 10-item scale concerning product labels (Q15), with measurements recorded on a 5-point Likert scale (1 = never to 5 = always). Following the preliminary exploratory factor analysis (EFA) and univariate comparisons within the framework of a multivariate analysis of covariance (MANCOVA), two representative items from question Q15 were selected as the primary dependent variables: the first regression model examined consumers’ purchase intentions when the product label explicitly states that it contains “selected premium-quality ingredients,” while the second regression model examined purchase intentions when the label specifies the product’s suitability “for a specific category of animals (e.g., for puppies/kittens, for neutered/spayed pets, for weight management).”

## 3. Results

### 3.1. Demographic Characteristics and Socioeconomic Status of Pet Owners

Regarding the demographic profile of the valid sample, clear structural patterns emerged in both pet ownership groups. In terms of gender distribution, a strong and statistically significant overrepresentation of female participants was observed. Women constituted the vast majority of the sample, representing 88.4% of dog owners (*n* = 220) and 93.1% of cat owners (*n* = 258), reflecting a demographic distribution that often aligns with contemporary research on the pet retail market, although caution is warranted regarding population-level generalizations due to potential self-selection patterns. The age distribution was heavily concentrated within the productive, middle-aged cohorts for both categories of owners. Specifically, the largest subgroup consisted of individuals aged 25–44 (52.2% for dog owners and 52.9% for cat owners), followed by the 45–64 age group (33.4% and 34.9%, respectively), while individuals aged 65 and over accounted for a small proportion (2.0% for both dog and cat owners). From a socioeconomic perspective, the reported net monthly household income indicated that the sample represented a stable, middle-class consumer base. Among the respondents who reported their household’s net monthly income in question Q30 (valid *n* = 219 for dog owners and *n* = 241 for cat owners, excluding participants who selected the response “Prefer not to answer”), the majority fell into the €501–€1000 bracket (44.7% for dog owners, 40.7% for cat owners) and the €1001–€2500 bracket (43.8% for dog owners, 44.8% for cat owners). The lowest-income group (≤€500) accounted for 6.4% of dog owners and 11.2% of cat owners, while the highest socioeconomic strata (>€2500) were at the opposite end of the distribution (5.0% and 3.3%, respectively). Behavioral and structural characteristics related to pets revealed fundamental variations between the two groups, providing a rich context for statistical contrast. In terms of pet density, single-pet ownership was largely dominant among dog owners (72.4%), while cat owners displayed a strong pattern of multiple-pet behavior, with only 37.4% owning a single cat and an impressive 25.2% managing more than three cats. In terms of breed orientation, mixed-breed animals constituted the absolute majority in both groups, although this tendency was much higher among cats (88.1%) compared to dogs (61.8%). The distribution of purebred animals was significantly more widespread among dog owners (28.7% versus 5.1% for cats). Furthermore, mean age profiles showed that the dog population was uniformly older across household ownership positions compared to the younger cat profile (e.g., the mean age of first pet ownership was 6.8 years for dogs versus 4.6 years for cats). Finally, veterinary health indicators and household care habits depicted a highly sophisticated, humanized care environment. Felines exhibited an exceptionally high rate of neutering (93.5% versus 69.7% for dogs), and both species lived primarily in secure, indoor domestic environments (84.0% for dogs and 89.5% for cats). Finally, the sample proved to be highly experienced, with 48.0% of dog owners and 36.8% of cat owners maintaining active guardianship for over a decade. The overall basic demographic and behavioral distributions of this sample are detailed in Table 2.

### 3.2. Product

In this subsection and the following ones, the presentation of the results has been organized based on the four Ps of the marketing mix.

The multivariate analysis (MANCOVA) framework employed in this section simultaneously evaluates a vector of 10 items related to product labels (Q15), while the subsequent multiple linear regression models isolate two specific outcome variables—measured on a 5-point Likert scale—pertaining to selected high-quality ingredients and specialized animal categories. This approach ensures that the primary outcome indicators, which capture purchasing behavior, are clearly defined.

A Multivariate Analysis of Covariance (MANCOVA) was conducted to evaluate consumer purchase intentions regarding a set of 10 items related to claims on product labels. The type of pet and net family income were used as independent categorical factors, while initial food selection criteria, attitudes toward the brand, and trust in the provided information were taken into account (as control variables) (Table 3). Regarding the multivariate and univariate diagnostic assumptions for MANCOVA, Box’s M test indicated that the observed covariance matrices of the dependent variables were not equal across groups (Box’s M = 426.11, F(275, 11,684.58) = 1.28, *p* = 0.001). Furthermore, Levene’s test for the equality of error variances indicated that the assumption of homoscedasticity was met for six of the ten dependent variables—specifically: “preservative-free” (F(7, 274) = 1.12, *p* = 0.348), “high protein content” (F(7, 274) = 0.74, *p* = 0.635), “low fat content” (F(7, 274) = 1.36, *p* = 0.223), “with natural preservatives” (F(7, 274) = 0.43, *p* = 0.881), “with selected high-quality ingredients” (F(7, 274) = 1.52, *p* = 0.161), and “hypoallergenic” (F(7, 274) = 1.00, *p* = 0.429)—whereas statistically significant violations were observed for: “grain-free” (F(7, 274) = 3.60, *p* = 0.001), “gluten-free” (F(7, 274) = 2.09, *p* = 0.044), “free from additives/preservatives” (F(7, 274) = 2.66, *p* = 0.011), and “for a specific category of animals” (F(7, 274) = 2.40, *p* = 0.021). Consequently, Pillai’s Trace (V) was used alongside Wilks’ Lambda (Λ), as the former is the most robust multivariate criterion against heterogeneity of variances and unequal group sizes; both statistics yielded identical F-values and significance levels for the main effects. The overall multivariate tests confirmed the existence of a statistically significant main effect for the factor “type of pet” (Pillai’s V = 0.073, Wilks’ Λ = 0.927, F(10, 247) = 1.955, *p* = 0.039, partial η^2^p = 0.073), demonstrating that dog and cat owners exhibit structurally distinct overall response profiles regarding product labeling. Among the control variables evaluated, significant multivariate predictive associations were identified for: perceived importance of ingredients (Pillai’s V = 0.138, Wilks’ Λ = 0.862, F(10, 247) = 3.939, *p* < 0.001, partial η^2^p = 0.138), influence of professional veterinary guidance (Pillai’s V = 0.084, Wilks’ Λ = 0.916, F(10, 247) = 2.278, *p* = 0.014, partial η^2^p = 0.084), and scientific substantiation of the brand (Pillai’s V = 0.094, Wilks’ Λ = 0.906, F(10, 247) = 2.577, *p* = 0.006, partial η^2^p = 0.094). No significant multivariate main effects were observed for net family income (*p* = 0.310, partial η^2^p = 0.043), nor for the interaction between animal species and income (*p* = 0.437, partial η^2^p = 0.040). Subsequently, follow-up univariate between-subjects tests were conducted to analyze the effects on each dependent variable (DV) individually, applying the Benjamini–Hochberg correction for the False Discovery Rate (FDR) across the set of 10 DVs. The results of these univariate analyses indicated that the criterion of ingredient quality significantly differentiated final purchase intentions for selected premium-category products, F(1, 256) = 6.938, *p* = 0.009 (FDR q = 0.013), partial η^2^p = 0.026, but had a negligible effect on specialized life-stage-specific foods, F(1, 256) = 0.004, *p* = 0.953 (FDR q = 0.953), partial η^2^p = 0.000. Conversely, professional veterinary recommendations significantly influenced intentions regarding specialized animal categories (life-stage/health-specific diets), F(1, 256) = 9.821, *p* = 0.002 (FDR q = 0.010), partial η^2^p = 0.037, whereas they did not show statistical significance regarding claims related to the use of premium-grade raw materials, F(1, 256) = 0.080, *p* = 0.777 (FDR q = 0.777), partial η^2^p = 0.000. Finally, the brand’s scientific substantiation significantly differentiated consumer response profiles concerning premium-grade formulations—F(1, 256) = 13.737, *p* < 0.001 (FDR q = 0.003), partial η^2^p = 0.051—and showed a marginal effect for specialized animal categories—F(1, 256) = 3.997, *p* = 0.047 (FDR q = 0.117), partial η^2^p = 0.015. These detailed metrics for each dependent variable (DV) provide the statistical basis for the multiple linear regression models that follow.

Table 4 presents the results of the multiple linear regression, with consumer purchasing behavior as the dependent variable in cases where a product label specifies the inclusion of premium-quality ingredients. The overall model was statistically significant, F(4, 464) = 37.64, *p* < 0.001, explaining 24.5% of the variance in the dependent variable (R^2^ = 0.245, Adjusted R^2^ = 0.238, Cohen’s f^2^ = 0.324). Comprehensive regression diagnostics confirmed that the model’s assumptions were met: the Durbin–Watson statistic (DW = 1.777) indicated the absence of autocorrelation among the residuals, while tolerance values (>0.789), VIF values (<1.267), and collinearity diagnostics (maximum Condition Index = 15.65) confirmed the absence of multicollinearity among the predictor variables. Examination of the standardized residuals (Mean = 0.00, Standard Deviation = 0.996), as well as the *ZRESID versus *ZPRED plots, confirmed the normality of the residuals and homoscedasticity.

All four predictor variables remained statistically significant after applying the Benjamini–Hochberg (FDR) correction to the model terms (all FDR q < 0.025). Specifically, the type of pet was a statistically significant predictor of purchasing behavior (B = 0.198, 95% CI [0.029, 0.366], β = 0.093, t = 2.309, *p* = 0.021, FDR q = 0.021), with cat owners showing a higher level of agreement regarding the purchase of products that explicitly mention specific, higher-quality ingredients, compared to dog owners.

Furthermore, the degree of influence exerted by ingredients when selecting the primary pet food emerged as the strongest predictor in the model (B = 0.404, 95% CI [0.298, 0.510], β = 0.340, t = 7.491, *p* < 0.001, FDR q < 0.001). Focusing on the standardized coefficients, the β value indicates that an increase of one standard deviation in the perceived importance of ingredients corresponds to an increase of 0.340 standard deviations in the intention to purchase premium-category products.

Furthermore, the degree of influence from a veterinarian’s recommendation was positively associated with purchasing behavior (B = 0.107, 95% CI [0.027, 0.187], β = 0.110, t = 2.631, *p* = 0.009, FDR q = 0.012). Finally, agreement with the statement that a pet food brand should present scientifically substantiated data also emerged as a statistically significant positive predictor (B = 0.162, 95% CI [0.085, 0.239], β = 0.184, t = 4.129, *p* < 0.001, FDR q < 0.001). To verify the robustness of the OLS (Ordinary Least Squares) estimates and to account for the ordinal nature of the outcome variable (a 5-point Likert scale), sensitivity analyses were conducted using a Generalized Linear Model (GLM) with robust standard errors, as well as an Ordinal Logistic Regression model (*n* = 469; with cat owners as the reference category, and coding: Dog = 1, Cat = 2). Both sensitivity analysis frameworks showed complete consistency—in terms of both direction and statistical significance—with the results obtained via the OLS method. Specifically, the GLM with robust standard errors was highly significant overall (Likelihood Ratio χ^2^(4) = 131.80, *p* < 0.001, Deviance/df = 0.855) and confirmed significant effects for the following variables: type of pet (B = −0.198, robust standard error = 0.087, 95% CI [−0.368, −0.027], Wald χ^2^(1) = 5.18, *p* = 0.023—confirming lower purchase intention among dog owners compared to cat owners), importance of ingredients (B = 0.404, robust standard error = 0.055, 95% CI [0.296, 0.512], Wald χ^2^(1) = 53.64, *p* < 0.001), veterinarian recommendation (B = 0.107, robust standard error = 0.042, 95% CI [0.025, 0.189], Wald χ^2^(1) = 6.53, *p* = 0.011), and scientific evidence (B = 0.162, robust standard error = 0.042, 95% CI [0.079, 0.244], Wald χ^2^(1) = 14.79, *p* < 0.001). Similarly, the Ordinal Logistic Regression model demonstrated a statistically significant fit (Likelihood Ratio χ^2^(4) = 144.59, *p* < 0.001, Deviance/df = 1.006), confirming that the type of pet (B = −0.377, SE = 0.175, 95% CI [−0.720, −0.033], Wald χ^2^(1) = 4.61, *p* = 0.032), the importance of ingredients (B = 0.879, SE = 0.119, 95% CI [0.646, 1.113], Wald χ^2^(1) = 54.38, *p* < 0.001), veterinary recommendation (B = 0.237, SE = 0.085, 95% CI [0.070, 0.404], Wald χ^2^(1) = 7.73, *p* = 0.005), and scientific substantiation (B = 0.341, SE = 0.081, 95% CI [0.183, 0.499], Wald χ^2^(1) = 17.82, *p* < 0.001) all remained strong predictors of consumer purchasing behavior regarding premium-quality ingredients.

Table 5 presents the results of the multiple linear regression, with consumer purchasing behavior as the dependent variable in cases where the product label indicates that it is intended for a specific category of animals (e.g., puppies/kittens, neutered/spayed animals, or animals on a weight management program). The overall model was statistically significant, F(4, 457) = 27.69, *p* < 0.001, explaining 19.5% of the variance in the dependent variable (R^2^ = 0.195, Adjusted R^2^ = 0.188, Cohen’s f^2^ = 0.242). Diagnostic checks confirmed the adequacy of the model: the Durbin–Watson statistic was 1.731; tolerance values (>0.792), VIF indices (<1.262), and collinearity diagnostics (maximum Condition Index = 15.65) confirmed the absence of multicollinearity; and the standardized residuals (Mean = 0.00, Standard Deviation = 0.996) satisfied the assumptions of normality and homoscedasticity. All four predictor variables remained statistically significant after the Benjamini–Hochberg FDR correction (all FDR q < 0.035). Specifically, the type of pet was a statistically significant predictor of purchasing behavior (B = 0.365, 95% CI [0.180, 0.549], β = 0.163, t = 3.879, *p* < 0.001, FDR q < 0.001), indicating that cat owners showed a stronger positive response to claims concerning specific animal categories compared to dog owners.

Furthermore, the degree of influence exerted by ingredients when selecting the primary pet food was positively associated with purchasing behavior (B = 0.127, 95% CI [0.011, 0.243], β = 0.101, t = 2.145, *p* = 0.032, FDR q = 0.032). Additionally, the degree of influence from a veterinarian’s recommendation emerged as a strong positive predictor (B = 0.219, 95% CI [0.131, 0.308], β = 0.211, t = 4.862, *p* < 0.001, FDR q < 0.001). Finally, agreement with the position that a food brand should present scientifically substantiated data emerged as the strongest predictor in the model (B = 0.243, 95% CI [0.159, 0.327], β = 0.262, t = 5.682, *p* < 0.001, FDR q < 0.001). Focusing on the standardized coefficients, an increase of one standard deviation in the demand for scientific substantiation corresponds to an increase of 0.262 standard deviations in purchase intentions for specialized products. In accordance with the analysis protocol, sensitivity analyses were also conducted for the outcome concerning the specific animal category (*n* = 462; reference category: Cat = 2, Dog = 1), using both a Generalized Linear Model (GLM) with robust standard errors and an Ordinal Logistic Regression model. Both models were highly significant overall (GLM: likelihood-ratio χ^2^(4) = 100.25, *p* < 0.001, deviance/degrees of freedom = 1.012; Ordinal Logistic Regression: likelihood-ratio χ^2^(4) = 104.78, *p* < 0.001, deviance/degrees of freedom = 1.082). These analyses strongly confirmed the initial findings from the OLS method for three of the four predictor variables: type of pet (GLM: B = −0.365, robust standard error = 0.095, 95% CI [−0.551, −0.178], Wald χ^2^(1) = 14.70, *p* < 0.001; Ordinal: B = −0.631, standard error = 0.179, 95% CI [−0.982, −0.281], Wald χ^2^(1) = 12.46, *p* < 0.001), veterinarian recommendation (GLM: B = 0.219, robust standard error = 0.045, 95% CI [0.132, 0.307], Wald χ^2^(1) = 24.05, *p* < 0.001; Ordinal: B = 0.442, standard error = 0.085, 95% CI [0.275, 0.609], Wald χ^2^(1) = 27.03, *p* < 0.001), and scientific evidence (GLM: B = 0.243, robust standard error = 0.049, 95% CI [0.147, 0.339], Wald χ^2^(1) = 24.69, *p* < 0.001; Ordinal: B = 0.496, standard error = 0.094, 95% CI [0.312, 0.679], Wald χ^2^(1) = 28.03, *p* < 0.001). Conversely, while the importance of ingredients was marginally significant in the parametric OLS model (*p* = 0.032), it did not reach the level of statistical significance (α = 0.05) when using robust variance estimation (GLM: B = 0.127, robust standard error = 0.074, 95% CI [−0.019, 0.272], Wald χ^2^(1) = 2.91, *p* = 0.088) or in an ordered data model (Ordinal: B = 0.213, standard error = 0.134, 95% CI [−0.050, 0.476], Wald χ^2^(1) = 2.52, *p* = 0.113). Although the positive direction of the coefficient remained consistent across all three approaches, the fact that zero is included within the confidence intervals—in both the robust estimation and the ordered data model—suggests that the direct effect of the general assessment of ingredients in the specialized pet food market (segmented by life stage) should be interpreted with caution. This further reinforces the conclusion that, when selecting specialized formulations, consumers rely primarily on scientific evidence and veterinary recommendations, rather than on an independent evaluation of the ingredients.

### 3.3. Price

According to Table 6, a one-way ANOVA was performed to investigate whether net income level is associated with differences in the criteria for choosing a staple food among dog and cat owners.

For dog owners, Levene’s test showed that the hypothesis of homogeneity of variance was satisfied for the criteria of price, ingredients, brand, advertising and packaging (*p* > 0.05). In contrast, a violation of the hypothesis was found for nutritional value, Levene’s F(3, 206) = 2.757, *p* = 0.043, as well as for veterinarian recommendation, Levene’s F(3, 200) = 4.096, *p* = 0.008. For this reason, in these cases, robust Welch tests were also examined, which did not reveal statistically significant differences for either nutritional value, Welch’s F(3, 30.950) = 1.199, *p* = 0.326, or for veterinarian recommendation, Welch’s F(3, 29.541) = 0.687, *p* = 0.567.

The results showed that there were no statistically significant differences between income groups in the importance attributed to price, F(3, 202) = 2.257, *p* = 0.083, ingredients, F(3, 203) = 0.911, *p* = 0.437, food brand, F(3, 197) = 0.669, *p* = 0.572, advertising, F(3, 193) = 0.677, *p* = 0.567, and packaging, F(3, 192) = 1.640, *p* = 0.182. Similarly, Welch tests confirmed that there were no statistically significant differences in nutritional value and veterinarian recommendation. Overall, the results suggest that monthly income level does not significantly affect dog owners’ food selection criteria.

For cat owners, Levene’s test showed that the assumption of homogeneity of variances was satisfied for all variables examined, as all significance values were greater than 0.05. Therefore, the ANOVA results are considered reliable without the need for using alternative robust tests.

The results of the analysis of variance (ANOVA) for cat owners showed no statistically significant differences between income groups regarding the importance of price, F(3, 229) = 1.138, *p* = 0.335, η^2^ = 0.015; ingredients, F(3, 226) = 0.242, *p* = 0.867, η^2^ = 0.003; nutritional value, F(3, 218) = 0.347, *p* = 0.791, η^2^ = 0.005; veterinary recommendation, F(3, 223) = 0.838, *p* = 0.474, η^2^ = 0.011; and packaging, F(3, 227) = 1.641, *p* = 0.181, η^2^ = 0.021. Conversely, prior to correction for multiple comparisons, differences were observed regarding the importance of food brand—F(3, 220) = 4.320, *p* = 0.006, η^2^ = 0.056 (Welch’s F(3, 29.83) = 4.384, *p* = 0.011)—as well as advertising—F(3, 214) = 3.121, *p* = 0.027, η^2^ = 0.042 (Welch’s F(3, 28.94) = 2.640, *p* = 0.068). Following the application of the Benjamini–Hochberg FDR procedure to the set of seven selection criteria for cat owners, the effect of income on the importance of the brand remained statistically significant (FDR q = 0.039), whereas the omnibus effect regarding advertising did not remain statistically significant after controlling for multiple comparisons (FDR q = 0.094). Bonferroni post-hoc analysis regarding the “brand” criterion confirmed that participants with a monthly income of €1001–€2500 (M = 3.6, SD = 1.0) placed significantly greater importance on the brand of pet food compared to those with an income of €501–€1000 (M = 3.1, SD = 1.2), with a mean difference of 0.470, 95% CI [0.039, 0.902], *p* = 0.025, and Cohen’s d = 0.452. No other pairwise comparisons between groups were found to be statistically significant. Regarding advertising, and consistent with the omnibus result following FDR adjustment, Bonferroni post-hoc tests did not reveal any statistically significant differences between specific pairs of income groups (all *p* > 0.05).

### 3.4. Place

According to Table 7, a one-way ANOVA was performed to investigate whether net income is associated with differences in pet food supply channels between dog and cat owners.

For dog owners, Levene’s test showed that the assumption of homogeneity of variance was satisfied for all examined supply channels, namely pet shops, supermarkets, veterinarians and online stores (*p* > 0.05). Therefore, the application of classical analysis of variance was deemed appropriate. The ANOVA results showed that there were no statistically significant differences between income groups in terms of the preference for purchasing food from pet shops, F(3, 196) = 0.659, *p* = 0.578, from supermarkets, F(3, 158) = 2.256, *p* = 0.084, from veterinarians, F(3, 163) = 0.012, *p* = 0.998, and through online stores, F(3, 171) = 0.812, *p* = 0.489. Descriptive statistics showed that the pet shop was the dominant supply channel regardless of income, with means ranging from 4.0 to 4.4. In contrast, purchasing from veterinarians and online showed moderate levels. Overall, the results suggest that the level of monthly income does not significantly affect the feed procurement channel for dog owners.

For cat owners, Levene’s test indicated that the assumption of homogeneity of variances was met for purchases from veterinarians (*p* = 0.609), but was violated for pet stores (*p* < 0.001), supermarkets (*p* = 0.010), and online stores (*p* < 0.001); consequently, robust Welch’s tests were examined alongside the classical analysis of variance (ANOVA). Application of the Benjamini–Hochberg false discovery rate (FDR) procedure across all four procurement channels confirmed that the three channels showing statistically significant results remained significant after correction for multiple comparisons. Specifically, no statistically significant differences were observed between income groups regarding the purchase of food from supermarkets, F(3, 185) = 1.534, *p* = 0.207, FDR q = 0.207, η^2^ = 0.024 (Welch’s F(3, 21.32) = 1.396, *p* = 0.271, FDR q = 0.271). Conversely, statistically significant differences were observed regarding purchases from pet stores, F(3, 213) = 11.278, *p* < 0.001, FDR q < 0.001, η^2^ = 0.137 (Welch’s F(3, 32.17) = 12.548, *p* < 0.001, FDR q < 0.001), from veterinarians, F(3, 193) = 5.200, *p* = 0.002, FDR q = 0.003, η^2^ = 0.075 (Welch’s F(3, 25.82) = 4.994, *p* = 0.007, FDR q = 0.011), as well as via online stores, F(3, 212) = 5.093, *p* = 0.002, FDR q = 0.003, η^2^ = 0.067 (Welch’s F(3, 24.41) = 4.910, *p* = 0.008, FDR q = 0.011). A Bonferroni post-hoc analysis revealed that, regarding acquisition from pet stores, individuals with an income of €1001–€2500 showed significantly lower levels of preference (M = 2.7, SD = 1.5) compared to both those with an income of €501–€1000 (M = 3.7, SD = 1.3)—mean difference = −1.017, 95% CI [−1.568, −0.466], *p* < 0.001, Cohen’s d = 0.712—and those with an income of up to €500 (M = 3.9, SD = 1.3)—mean difference = −1.256, 95% CI [−2.061, −0.451], *p* < 0.001, Cohen’s d = 0.852. Regarding acquisition through a veterinarian, participants with an income of €1001–€2500 showed a significantly higher preference (M = 3.3, SD = 1.3) compared to those with an income of €501–€1000 (M = 2.5, SD = 1.4), with a mean difference of 0.812, 95% CI [0.244, 1.379], *p* = 0.001, Cohen’s d = 0.592. Regarding online shopping, the group with an income of €1001–€2500 also showed a significantly higher preference (M = 3.8, SD = 1.1) compared to the group with an income of €501–€1000 (M = 3.1, SD = 1.5), with a mean difference of 0.703, 95% CI [0.165, 1.241], *p* = 0.004, Cohen’s d = 0.532. The remaining pairwise comparisons were not statistically significant.

### 3.5. Promotion

In Table 8, both parametric (Pearson’s r) and non-parametric (Spearman’s rho) bivariate correlations were examined to assess whether label characteristics that influence product purchasing are associated with interest in specific types of advertising. Both correlation methods showed a high degree of agreement. Even in cases where correlations were statistically significant—including after applying the Benjamini–Hochberg False Discovery Rate (FDR) procedure to the set of 50 correlations per animal category—the strength of these associations remained consistently weak; all Pearson coefficients fell below the |0.30| threshold (while Spearman’s rho reached a maximum of 0.314 for cats, in the context of specific animal categories and posts featuring pets). This confirms that preferences regarding the format of promotional media and the evaluation of nutritional labels function, to a large extent, independently of one another.

Table 9 presents the parametric (Pearson’s r) and non-parametric (Spearman’s rho) correlations between the level of trust in information sources and the types of advertisements that are of greatest interest to dog and cat owners. All reported correlations, with an absolute effect size greater than |0.30|, remained highly significant (FDR q < 0.01) following the Benjamini–Hochberg correction for multiple comparisons (applied to 25 tests per animal species), while the Spearman rank correlations confirmed the exact same patterns in terms of direction and magnitude.

For the dog category, trust in advertisements showed a moderate positive correlation with interest in photographs (r = 0.314, rho = 0.310, *p* < 0.001, FDR q < 0.001) and in content creators (r = 0.319, rho = 0.335, *p* < 0.001, FDR q < 0.001). Trust in influencers showed a strong positive correlation with advertisements featuring influencers (r = 0.548, rho = 0.574, *p* < 0.001, FDR q < 0.001) and a moderate-to-strong correlation with content creators (r = 0.457, rho = 0.468, *p* < 0.001, FDR q < 0.001). Furthermore, trust in pet stores showed a moderate positive correlation with photographs (r = 0.341, rho = 0.331, *p* < 0.001, FDR q < 0.001). Additionally, trust in social media showed a moderate positive correlation with advertisements involving influencers (r = 0.344, rho = 0.366, *p* < 0.001, FDR q < 0.001), as well as with content creators (r = 0.397, rho = 0.400, *p* < 0.001, FDR q < 0.001). Regarding the cat category, trust in veterinarians showed a strong positive correlation with posts featuring pets (r = 0.541, rho = 0.542, *p* < 0.001, FDR q < 0.001), while a moderate to strong negative correlation was observed with content creators (r = −0.417, rho = −0.416, 95% CI [−0.511, −0.314], *p* < 0.001, FDR q < 0.001). Trust in advertisements showed a strong positive correlation with posts featuring pets (r = 0.502, rho = 0.539, *p* < 0.001, FDR q < 0.001) and a moderate positive correlation with advertisements by influencers (r = 0.408, rho = 0.439, *p* < 0.001, FDR q < 0.001). At the same time, trust in influencers showed a moderate positive correlation with photographs (r = 0.434, rho = 0.451, *p* < 0.001, FDR q < 0.001) and a strong positive correlation with content creators (r = 0.648, rho = 0.662, *p* < 0.001, FDR q < 0.001), while a moderate negative correlation was observed with posts featuring pets (r = −0.321, rho = −0.312, 95% CI [−0.423, −0.211], *p* < 0.001, FDR q < 0.001). Furthermore, trust in pet stores showed a strong positive correlation with posts featuring pets (r = 0.550, rho = 0.545, *p* < 0.001, FDR q < 0.001). Finally, trust in social media showed moderate to strong positive correlations with photographs (r = 0.341, rho = 0.351, *p* < 0.001, FDR q < 0.001), posts featuring pets (r = 0.414, rho = 0.422, *p* < 0.001, FDR q < 0.001), advertisements by influencers (r = 0.552, rho = 0.541, *p* < 0.001, FDR q < 0.001), and content creators (r = 0.465, rho = 0.478, *p* < 0.001, FDR q < 0.001).

Regarding the baseline frequencies of exposure to advertisements through various communication channels (Q19: television, the internet, social media, pet stores, and print media), preliminary one-way analyses of variance (ANOVAs)—based on household net income brackets—did not reveal any statistically significant differences for either dog owners or cat owners (in all cases, *p* > 0.05). These findings suggest a uniform level of baseline exposure across all socioeconomic strata, with a primary emphasis on digital media and social media.

A one-way analysis of variance (ANOVA) was conducted to examine whether the level of monthly income is associated with differences in the degree of interest in various types of advertisements among dog owners (Table 10). Prior to performing the ANOVA, the assumption of homogeneity of variances was tested using Levene’s test. The results indicated that the assumption of equal variances was met for all forms of advertising, as all significance values were greater than 0.05. The ANOVA results for dog owners indicated no statistically significant differences between income groups regarding interest in video-based advertisements, F(3, 205) = 0.373, *p* = 0.773; photographs, F(3, 198) = 0.237, *p* = 0.871; posts featuring pets, F(3, 203) = 0.246, *p* = 0.864; influencer-led advertisements, F(3, 194) = 0.630, *p* = 0.596; or content from content creators, F(3, 195) = 0.523, *p* = 0.667. Descriptive statistics showed relatively similar means across all income groups. Participants with a monthly income of up to €500 exhibited slightly higher levels of interest in most forms of advertising—notably for videos (M = 3.6, SD = 1.0) and posts featuring pets (M = 3.3, SD = 1.3)—compared to individuals in higher income brackets; however, these differences were small and not statistically significant. These parametric findings were further supplemented by non-parametric Kruskal–Wallis H tests, which likewise revealed no statistically significant differences among income categories for dog owners regarding their interest in videos (χ^2^(3) = 0.852, *p* = 0.837), photographs (χ^2^(3) = 0.685, *p* = 0.877), pet-related posts (χ^2^(3) = 0.663, *p* = 0.882), advertisements by influencers (χ^2^(3) = 1.938, *p* = 0.585), or content from content creators (χ^2^(3) = 1.879, *p* = 0.598). Overall, both the parametric and non-parametric results confirm that the level of monthly income does not significantly influence dog owners’ preferences regarding various types of advertising content. Regarding the cat-related data, Levene’s test indicated that the assumption of homogeneity of variances was met for most variables (*p* > 0.05), with the exception of interest in content from content creators, where a violation of the assumption was observed: Levene’s F(3, 224) = 3.196, *p* = 0.024. Consequently, robust Welch tests were also conducted, which confirmed the results of the analysis of variance (ANOVA). The findings showed no statistically significant differences between income groups regarding interest in video-based advertisements, F(3, 226) = 0.291, *p* = 0.832, or in advertisements featuring influencers, F(3, 218) = 1.251, *p* = 0.292.

Conversely, for cat owners, statistically significant differences were observed regarding interest in advertisements featuring photographs, F(3, 223) = 3.460, *p* = 0.017, FDR q = 0.029, η^2^ = 0.044; in posts featuring pets, F(3, 222) = 3.568, *p* = 0.015, FDR q = 0.029, η^2^ = 0.046; and in content from content creators, F(3, 224) = 5.548, *p* = 0.001, FDR q = 0.005, η^2^ = 0.069. All these differences remained statistically significant across all five advertisement formats following the application of the Benjamini–Hochberg FDR procedure. The results were also confirmed via Welch’s tests: for photographs, Welch’s F(3, 28.64) = 3.242, *p* = 0.037; for posts featuring pets, Welch’s F(3, 28.63) = 2.978, *p* = 0.048; and for content creators, Welch’s F(3, 28.57) = 5.419, *p* = 0.004. Furthermore, a non-parametric sensitivity analysis, using the Kruskal–Wallis H test, fully corroborated these results, demonstrating statistically significant differences across income brackets regarding photographs (χ^2^(3) = 12.153, *p* = 0.007), posts featuring pets (χ^2^(3) = 10.463, *p* = 0.015), and content creators (χ^2^(3) = 16.337, *p* = 0.001), while also confirming the absence of statistical significance for videos (χ^2^(3) = 1.719, *p* = 0.633) and influencer advertisements (χ^2^(3) = 4.998, *p* = 0.172). However, a post-hoc analysis using the Bonferroni correction revealed that the statistically significant differences between the groups were localized to specific comparisons based on income. Specifically, regarding advertisements that included photographs, individuals with a monthly income of €1001–€2500 showed significantly higher interest (M = 3.2, SD = 1.2) compared to those with an income of €501–€1000 (M = 2.7, SD = 1.0), with a mean difference of 0.459, 95% CI [0.035, 0.884], *p* = 0.026, and an effect size of Cohen’s d = 0.452. Regarding posts that included pets, although the overall analysis of variance (ANOVA) was statistically significant, Bonferroni post-hoc comparisons did not reveal statistically significant differences between specific income groups (all *p* > 0.05), suggesting that the variation between the groups ceased to be statistically significant following the application of this conservative post-hoc correction. Finally, regarding interest in content from content creators, the group with an income of €1001–€2500 showed significantly higher levels of interest (M = 2.9, SD = 1.2) compared to both the €501–€1000 income group (M = 2.3, SD = 1.1; mean difference = 0.600, *p* = 0.005, Cohen’s d = 0.521) and the group with an income of up to €500 (M = 2.0, SD = 1.2; mean difference = 0.900, *p* = 0.008, Cohen’s d = 0.814). No other statistically significant differences were observed among the remaining income groups.

## 4. Discussion

The demographic profile of the survey participants is dominated by women, highlighting a distinct demographic pattern within the group under study—one that aligns with international research on pet care [34,52]—while necessitating a careful distinction between the sample’s gender composition and the monitoring of actual household purchasing habits (88.4% for dogs and 93.1% for cats) and middle-class owners a structural distribution extensively documented in the international literature on pet care and the agri-food sector, reflecting a known pattern of digital self-selection rather than a sampling anomaly. In addition, there is a strong overrepresentation of pets that live at home and are neutered, which is particularly important as it likely indicates guardians with a high interest in their pet’s nutrition and high involvement in purchasing behavior (see Table 2).

The incorporation of the comprehensive MANCOVA framework (Table 3) provides a robust basis for the study’s findings, demonstrating that the type of companion animal—alongside key behavioral control variables—systematically determines how pet owners process pet food labels. Overall, the regression results in Table 4 suggest that purchasing behavior towards products featuring selected premium ingredients is positively influenced by the type of pet (*β* = 0.093, *p* = 0.021), the importance attributed to food ingredients (*β* = 0.340, *p* < 0.001) the influence of veterinarian recommendation (*β* = 0.110, *p* = 0.009), and the need for scientifically substantiated information (*β* = 0.184, *p* < 0.001). Among all factors, the importance of ingredients emerged as the strongest predictor of consumer behavior (*t* = 7.491, *p* < 0.001). The importance of ingredient evaluation is in line with Ai et al. [34], who identify formulation transparency as one of the main factors in pet food selection in the modern era. Furthermore, the variation recorded by species reflects the specialized and selective feeding behaviors of felines, which cause cat owners to prioritize high-end formulations more strongly than dog owners, as documented by Willoughby et al. [52]. The simultaneous importance of veterinary guidance and scientific data highlights a growing shift among the examined Greek respondents towards professional validation and empirical support over commercial marketing claims, a behavioral trend confirmed by Jobe & Downs [24].

It is worth highlighting that a comparative analysis between the two regression models reveals a significant shift in consumer priorities. While general claims for premiums (Table 4) are mainly driven by component evaluation, purchases targeting specific health categories or life stages (Table 5) depend heavily on professional and empirical validation. This finding suggests that the more importance consumers attach to food ingredients, the more likely they are to be positively influenced by the mention of premium ingredients on the label. Table 5 shows that buying decisions towards products that promote their suitability for specific animal categories is positively influenced by the type of pet (*β* = 0.163, *p* < 0.001), the importance attributed to food ingredients (*β* = 0.101, *p* = 0.032), the influence of the veterinarian’s recommendation (*β* = 0.211, *p* < 0.001) and the need for scientifically substantiated information (*β* = 0.262, *p* < 0.001). It is worth noting that, while the importance attributed to food ingredients showed a moderate positive correlation in the initial linear model (*p* = 0.032), sensitivity analyses—using Generalized Linear Models with robust standard errors (*p* = 0.088) and Ordinal Logistic Regression (*p* = 0.113)—indicated that this effect ceases to be statistically significant under more conservative specifications (which account for ordinal data and are robust to heteroscedasticity). In contrast, the type of pet, veterinary recommendations, and scientific substantiation remained highly stable parameters (*p* < 0.001 across all models). This discrepancy further reinforces the conclusion that, when owners select specialized diets for specific life stages or health conditions, the independent evaluation of ingredients becomes secondary, taking a back seat to professional clinical guidance and the scientific foundation provided by the company itself. Of all the factors examined, the importance of the brand’s empirical documentation emerged as the strongest predictor of this specific consumer response (*t* = 5.682, *p* < 0.001). The prominence of scientific support over simple marketing claims is strongly highlighted by Ashwell et al. [53], who highlighted that modern pet guardians demand objective and empirical evidence before trusting claims for specialty foods, including pet food. This result suggests that consumers who place greater emphasis on the scientific substantiation of the information provided by a company are more likely to trust and choose products that are addressed to specific animal categories. Similarly, the strong influence of veterinary recommendations aligns with the research of Scarpim & Pacheco [19], confirming that expert clinical consultation remains a fundamental catalyst when pet owners seek formulas for different life segments or health conditions (e.g., puppy or weight management). The variation by species, showing higher compliance among cat owners, reflects the notoriously fickle nutritional habits characteristic of felines, which inevitably force their owners to seek specialized, targeted diets more intensively than dog owners [54].

According to Table 6, the results suggest that the level of monthly income is associated with a limited extent to the food selection criteria of cat owners, mainly linked to the importance attributed to the food brand (F(3, 220) = 4.320, *p* = 0.006) and advertising preferences (F(3, 214) = 3.12, *p* = 0.027), with cat owners belonging to the highest-income subgroup (*n* = 8) recording a higher descriptive mean regarding advertising (M = 4.0, SD = 1.6), although Bonferroni post-hoc tests did not confirm significant differences between the individual income brackets. Conversely, criteria such as price, ingredients, nutritional value, professional endorsement by veterinarians, and packaging do not show significant differences across socioeconomic strata (*p* > 0.05 in all cases). This limited influence of socioeconomic status on feline dietary choices demonstrates that modern pet owners prioritize nutritional value and food safety as non-negotiable standards, regardless of household income variation [21]. However, the variation recorded in brand importance and advertising receptivity between middle- to high-income segments reflects a well-documented trend in consumer behavior in pet retail. Higher-income pet owners often view established premium brands and targeted media messaging as the primary symbol of quality and safety assurance [55,56].

Table 7 suggests that the level of monthly income is related to certain channels of pet food procurement for cat owners. In particular, people with an income of €1001–2500 seem to prefer procurement through veterinarians (F(3, 193) = 5.200, *p* = 0.002) and online stores more (F(3, 212) = 5.093, *p* = 0.002), while they show a lower preference for purchases from pet shops (F(3, 213) = 11.278, *p* < 0.001) compared to some other income categories. In contrast, procurement from supermarkets does not differ significantly between income groups (F(3, 185) = 1.534, *p* = 0.207). This particular consumer shift among middle- to high-income cat owners, from traditional retail to specialized or digital channels, reflects the broader market dynamics documented by Wang & Jia [57], who highlight that higher disposable income is strongly associated with a higher preference for shopping convenience and variety of high-quality products found online. At the same time, the results reveal that there is an increased reliance on veterinary supplies at this socioeconomic level. Middle- to high-income pet owners often demonstrate a stronger tendency to incorporate professional clinical advice directly into their regular purchases, prioritizing medical validation over commercial outlets [34,58,59,60]. In contrast, the stable, non-significant preference for the supermarket distribution channel confirms that the procurement of animal feed from such stores is primarily seen as a standard convenience base that operates regardless of income level [61,62].

The analysis in Table 8 shows that, although several correlations are statistically significant, their strength remains consistently weak, with all coefficients remaining below the 0.30 threshold. This suggests that the sampled owners’ preference for specific forms of advertising (e.g., videos, photos, or influencer posts) is not strongly associated with the nutritional claims that guide their purchases [63,64,65]. Evaluating health claims is a cognitive process focused on pet health, while preference for media format is driven by entertainment habits [24,65]. Pet owners seem to separate marketing techniques from reading labels based on factual information. An attractive post may create brand awareness, but it plays little role in critically evaluating nutritional claims [66,67,68].

The results of Table 9 show that trust in more digital and socially oriented information sources is more strongly associated with interest in modern forms of advertising content, with correlations reaching up to *r* = 0.548 for dog owners and *r* = 0.648 for cat owners (both *p* < 0.01). This interaction suggests that higher perceived digital credibility is closely linked to increased receptiveness toward the corresponding marketing channels, without, however, implying a definitive causal relationship regarding directionality [69].

For dog owners, Table 10 suggests that monthly income level does not significantly influence preferences for any type of advertising content (all *p* > 0.05). In contrast, for cat owners, monthly income level is mainly associated with interest in photos (F(3, 223) = 3.460, *p* = 0.017) and content from content creators (F(3, 224) = 5.548, *p* = 0.001), while the remaining advertising formats do not appear to differ significantly across income categories (all *p* > 0.05). Cat owners often present highly specialized and diverse digital consumer profiles compared to dog owners, rendering them more closely linked to targeted aesthetic changes across all socioeconomic segments [70,71].

**Figure 3:**
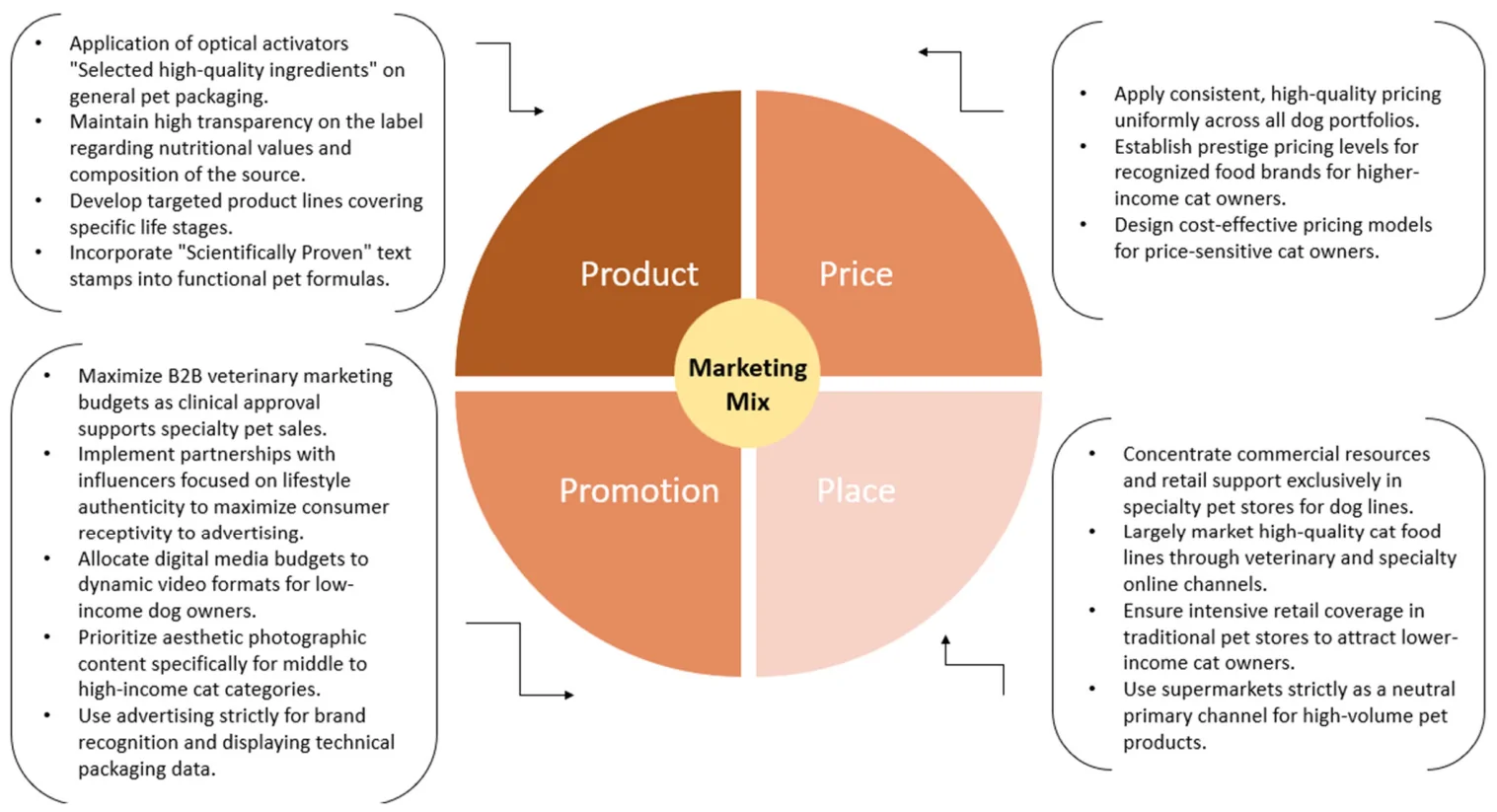
Proposed strategic Marketing Mix (4Ps) for the dog and cat food industry.

Taking into account the empirical findings and statistical analysis of the research, a targeted marketing mix was developed for businesses active in the production and marketing of pet food. This framework aims to provide operational and practical guidance for managers in the industry. The proposed marketing strategy is illustrated in Figure 3.

Despite the valuable strategic insights that emerge from this study, there are some limitations. First, the survey was based on snowball sampling and was conducted online, which may limit the generalizability of the results to the broader population of dog and cat owners. In particular, online distribution may sometimes represent primarily younger individuals with a good relationship with technology, resulting in them being more exposed to digital advertising content. In addition, due to the snowball sampling technique, survey participants may have been clustered, sharing a similar profile in pet care. Finally, the cross-sectional design of the survey captures the consumer attitudes of dog and cat owners for the specific time point when the survey was conducted and the primary data were collected. On the other hand, the pet food industry is evolving rapidly, making it impossible to capture consumer behavior in the long term. Furthermore, certain limitations regarding the sample characteristics should be acknowledged. First, due to strict anonymity protocols and the voluntary nature of the digital distribution, specific geographical location data within Greece were not collected from participants; however, the online sampling approach—utilizing nationwide pet owner communities—ensures national reach and representativeness. Second, the final sample exhibits a marked gender imbalance, with a predominance of women (88.4% for dog owners and 93.1% for cat owners). Although contemporary literature often links this demographic pattern to the role of women as the primary individuals responsible for household pet nutrition, this distribution also reflects a well-documented self-selection bias, wherein women demonstrate higher participation rates in voluntary online surveys concerning lifestyle and animal welfare. Consequently, the results cannot be generalized, beyond the sample groups; furthermore, variations in baseline characteristics—such as neutering rates, pet density, and breed distribution—reflect deeply intertwined lifestyle patterns characterizing each population of pet owners. This implies that the observed behavioral differences reflect the overall consumer profile of dog guardians compared to that of cat guardians, rather than merely the isolated effect of the animal species—a factor that should be taken into account in future multivariate stratification designs; additionally, because the consumer measurements were reported entirely by the consumers themselves, the findings may be subject to potential recall bias and social desirability bias, particularly regarding sensitive economic variables such as self-reported monthly income brackets and food expenditures. The potential lack of independence between owners of multiple pets and household budget configurations must also be considered an inherent design limitation. Furthermore, given that all predictor and outcome variables were collected simultaneously via a single self-report questionnaire, the data may be subject to common method bias (CMB). Moreover, the survey items assessed self-reported attitudes and hypothetical purchase intentions rather than objectively recorded retail transactions; consequently, when interpreting the practical implications for marketing, potential gaps between intention and behavior should be taken into account. Finally, although the application of the Benjamini–Hochberg procedure for controlling the False Discovery Rate (FDR), Bonferroni (post-hoc) corrections, and non-parametric sensitivity analyses mitigated the risk of an inflated Type I error rate when conducting multiple statistical comparisons, the exploratory correlations emerging from this cross-sectional study—particularly within smaller subgroups with extreme income levels—should be interpreted with due caution and require further validation in future prospective studies that incorporate stratification.

## 5. Conclusions

This study included a comprehensive empirical examination of the impact of dog and cat food advertisements, packaging claims, and various trust factors on the purchasing behavior of pet owners. Statistical analysis on a sample of 526 valid responses provides empirical evidence consistent with the underlying research hypotheses, demonstrating that dog and cat food purchasing is not only driven by basic nutritional knowledge but also by strong nutritional awareness, the search for professional validation, and the individual dynamics of consumers by pet species. Regression models reveal a shift in consumer preferences depending on the type and category of pet food. In premium formulas, survey participants appear to be cognitively engaged with the product, placing greater emphasis on the selected ingredients of pet recipes. However, when the consumer is interested in specialized food categories depending on the life stage of the pet, the influence of reading the main ingredients of the product decreases. In this case, dog and cat owners give great weight to the scientific data of the brand as well as to the recommendations of specialized professionals, such as veterinarians. In addition, this study reveals critical behavioral deviations, which seem to be related to the type of pet as well as the socioeconomic profile of the pet owner. The strategic marketing mix resulting from this research can provide guidelines to businesses operating in the examined sector in order to more effectively manage the retail sale of dog and cat food. Optimizing these corporate strategies could serve a dual purpose: enhancing product transparency for the consumer while simultaneously supporting more rational nutritional decision-making under veterinary guidance, thereby ensuring the long-term nutritional health and well-being of companion animals.

### 5.1. Implications of the Study

#### 5.1.1. Theoretical Implications

The study has theoretical implications since it enriches the theory of the humanization of pets, revealing that the purchase of dog and cat food has shifted from a low-involvement act to a major decision determining the health of the animal. The processing of information about the food products of the examined pets can lead the consumer’s behavior to either independently evaluate the ingredients or to submit to the opinion of the industry professional who provides scientific documentation. Moreover, the particular biological characteristics of felines seem to directly influence the consumer behavior of their owner, a pattern that is completely absent among dog owners.

#### 5.1.2. Practical Implications

In terms of practical implications, dog and cat food companies should focus on the transparency of raw materials and the use of high-quality ingredients, which should be scientifically documented. The significant role of veterinarians, as highlighted in this study, underscores the need to utilize this scientific evidence to develop comprehensive information programs for pet owners that will ensure the rational nutrition of companion animals in the long term. The distribution channels used should be reconfigured, since dog owners seem to have a strong overall preference for specialized pet stores, while on the other hand the sale of premium cat food should be focused on veterinary and digital sites for those higher-income consumer segments. The shift of distribution channels towards clinics and professional platforms is likely to contribute to establishing better communication between professionals and pet owners. Furthermore, cross-promotional budgets should correspond to the demographic profiles of consumers, emphasizing dynamic video content across dog-owning profiles and photographic content for affluent cat owners. Adapting to these communication channels can help animal feed companies and veterinarians more effectively convey targeted messages—leveraging specific scientific data on animal nutrition and welfare—to different consumer segments.

#### 5.1.3. Policy Implications

Our study findings reveal that vague or unverified claims concerning various animal life stages significantly distort owners’ perceptions. From a regulatory perspective, responsible regulatory bodies should establish strict definitions for vaguely broad marketing terms used in pet foods, such as premium or selected ingredients, in order to protect consumers from being misled. This highlights the critical importance of empirical and scientific validation of foods promoted for specific biological stages and phases of the animal’s life. In accordance with Regulation (EC) No 767/2009 on the placing on the market and use of feed, marketing claims regarding the composition, properties, and functions of pet food must be objective, verifiable, and scientifically substantiated.

## Figures and Tables

**Table 3:** Multivariate analysis of covariance (MANCOVA) results for pet owner purchasing behavior across label claims.

Effect	Pillai’s Trace (V)	Wilks’ Lambda (Λ)	F	Hypothesis df	Error df	*p*-Value	Partial η^2^p
Companion animal species	0.073	0.927	1.955	10.000	247.000	0.039	0.073
Importance of ingredients	0.138	0.862	3.939	10.000	247.000	<0.001	0.138
Veterinarian recommendation	0.084	0.916	2.278	10.000	247.000	0.014	0.084
Importance of scientific data	0.094	0.906	2.577	10.000	247.000	0.006	0.094
Monthly net income	0.129	0.876	1.114	30.000	725.670	0.310	0.043
Species X Income Interaction	0.117	0.886	1.021	30.000	725.670	0.437	0.040

**Table 4:** Multiple Linear Model for Predicting Consumer Buying Behavior When the Product Label Mentions Selected Premium Ingredients.

Model		Unstandardized Coefficients		95% CI for B [LL, UL]	Standardized Coefficients	*t*	*p*	Collinearity Statistics	
		*B*	Std. Error		*β*			Tolerance	VIF
1	(Constant)	0.746	0.266	[0.223, 1.268]		2.805	0.005		
	Pet (1 = Cat, 0 = Dog)	0.198	0.086	[0.029, 0.366]	0.093	2.309	0.021	0.995	1.005
	Degree of influence of ingredients on the choice of main food for the pet	0.404	0.054	[0.298, 0.510]	0.340	7.491	0.000	0.789	1.267
	Degree of influence from a veterinarian’s recommendation for choosing a main food for the pet	0.107	0.041	[0.027, 0.187]	0.110	2.631	0.009	0.934	1.070
	Degree of agreement on whether it is important for a food brand to present scientifically substantiated data	0.162	0.039	[0.085, 0.239]	0.184	4.129	0.000	0.823	1.215

**Table 5:** Multiple Linear Model for Predicting Consumer Buying Behavior When the Product Label Mentions the Specific Animal Category.

Model		Unstandardized Coefficients		95% CI for B [LL, UL]	Standardized Coefficients	*t*	*p*	Collinearity Statistics	
		*B*	Std. Error		Beta			Tolerance	VIF
1	(Constant)	1.214	0.291	[0.642, 1.787]		4.169	0.000		
	Pet (1 = Cat, 0 = Dog)	0.365	0.094	[0.180, 0.549]	0.163	3.879	0.000	0.994	1.006
	Degree of influence of ingredients on the choice of main food for the pet	0.127	0.059	[0.011, 0.243]	0.101	2.145	0.032	0.792	1.262
	Degree of influence from a veterinarian’s recommendation for choosing a main food for the pet	0.219	0.045	[0.131, 0.308]	0.211	4.862	0.000	0.931	1.074
	Degree of agreement on whether it is important for a food brand to present scientifically substantiated data	0.243	0.043	[0.159, 0.327]	0.262	5.682	0.000	0.826	1.211

**Table 6:** Differences between income groups in terms of food selection criteria and type of pet (dogs vs. cats).

		Monthly Net Income (€):							
		Over 2500		1001–2500		501–1000		Up to 500	
		M	SD	M	SD	M	SD	M	SD
Dog	Price	2.7	1.5	2.9	1.1	3.3	1.2	3.1	1.4
	Ingredients	4.5	0.8	4.0	0.9	4.1	0.8	4.0	0.6
	Nutritional value	4.5	0.5	4.2	0.7	4.2	0.8	4.1	0.6
	Brand	3.1	1.1	3.0	1.3	2.9	1.1	3.4	1.2
	Veterinarian recommendation	4.0	0.7	3.7	1.2	3.6	1.1	3.6	0.9
	Advertising	1.5	0.5	1.7	0.8	1.8	0.9	2.0	1.1
	Packaging (e.g., photo, ecological, practical)	1.5	0.5	1.7	0.8	2.0	0.9	1.9	1.1
Cat	Price	2.9	1.2	3.4	1.0	3.5	1.2	3.7	1.1
	Ingredients	3.9	1.0	4.1	0.8	4.1	0.9	4.1	1.0
	Nutritional value	4.0	0.9	3.7	1.0	3.7	1.2	3.9	1.1
	Brand	3.9	1.0	3.6	1.0	3.1	1.2	3.0	1.1
	Veterinarian recommendation	3.5	1.2	3.8	1.1	3.6	1.0	3.5	1.0
	Advertising	4.0	1.6	2.9	1.4	3.3	1.2	3.5	1.3
	Packaging (e.g., photo, ecological, practical)	2.8	1.4	2.2	1.0	2.5	1.1	2.3	1.1

**Table 7:** Differences between incomes in terms of supply channels and type of pet (Dogs, cats).

		Monthly Net Income (€):							
		Over 2500		1001–2500		501–1000		Up to 500	
		M	SD	M	SD	M	SD	M	SD
Dog	Pet shop	4.4	1.0	4	1.1	4	1.2	4.2	0.7
	Supermarket	2.4	1.6	1.8	1.1	1.9	1.2	2.7	1.5
	Veterinary	2.7	1.4	2.8	1.4	2.8	1.4	2.8	1.2
	Online	2.5	1.4	2.9	1.4	2.5	1.5	2.4	1.6
Cat	Pet shop	4	0.6	2.7	1.5	3.7	1.3	3.9	1.3
	Supermarket	3	1.4	2.8	1.1	2.4	1.4	2.4	1.2
	Veterinary	2.9	1.2	3.3	1.3	2.5	1.4	2.6	1.5
	Online	2.6	1.7	3.8	1.1	3.1	1.5	3.3	1.6

**Table 8:** Correlation of the Label Features that drive the purchase of the product with the Advertisements that show the greatest interest.

**Dogs** **Features/Advertisements**	**Video**	**Photo**	**Post with Pets**	**Influencers**	**Content Creators**
Without preservatives	0.075 (0.060)	0.083 (0.099)	0.103 (0.097)	0.046 (−0.001)	0.119 (0.069)
High protein	0.028 (−0.003)	0.109 (0.095)	0.076 (0.048)	0.049 (0.013)	0.044 (0.013)
Low fat	0.152 * (0.128)	0.147 * (0.137 *)	0.219 ** (0.189 **)	0.052 (0.026)	0.060 (0.032)
With natural preservatives	0.107 (0.068)	0.236 ** (0.229 **)	0.181 ** (0.165 *)	0.082 (0.072)	0.157 * (0.135 *)
With selected premium ingredients	0.083 (0.058)	0.129 (0.134 *)	0.100 (0.094)	0.051 (0.040)	0.081 (0.062)
Grain-free	0.035 (0.035)	0.075 (0.073)	0.070 (0.043)	0.025 (0.057)	0.037 (0.058)
Gluten free	0.000 (−0.002)	0.072 (0.084)	0.056 (0.070)	0.038 (0.069)	0.055 (0.091)
No additives/preservatives	0.020 (−0.002)	0.024 (0.026)	0.067 (0.054)	0.026 (0.076)	0.122 (0.147^*^)
Hypoallergenic	0.105 (0.101)	0.079 (0.108)	0.149 * (0.152 *)	0.175 * (0.181 **)	0.159 * (0.149 *)
For a special category of animal (e.g., for puppies, for neutered animals, for weight control)	0.076 (0.092)	0.097 (0.109)	0.088 (0.076)	0.093 (0.074)	0.132 (0.126)
**Cats** **Features/Advertisements**	**Video**	**Photo**	**Post with Pets**	**Influencers**	**Content Creators**
Without preservatives	0.111 (0.129 *)	0.192 ** (0.216 **)	−0.054 (−0.108)	−0.008 (−0.049)	0.241 ** (0.257 **)
High protein	−0.082 (−0.108)	−0.109 (−0.138*)	0.293 ** (0.296 **)	0.100 (0.085)	−0.070 (−0.100)
Low fat	−0.136 * (−0.168 **)	−0.086 (−0.102)	0.034 (0.032)	0.053 (0.069)	−0.045 (−0.062)
With natural preservatives	−0.006 (−0.035)	0.131 * (0.120)	0.039 (0.042)	0.125 (0.137 *)	0.002 (−0.007)
With selected premium ingredients	0.036 (0.025)	0.088 (0.092)	0.062 (0.058)	0.055 (0.027)	0.092 (0.069)
Grain-free	−0.020 (−0.026)	−0.013 (−0.017)	0.245 ** (0.241 **)	0.043 (0.023)	−0.060 (−0.068)
Gluten free	−0.063 (−0.063)	0.065 (0.075)	−0.096 (−0.099)	0.031 (0.037)	0.128 * (0.128 *)
No additives/preservatives	0.053 (0.054)	0.056 (0.060)	0.103 (0.079)	−0.092 (−0.110)	0.077 (0.078)
Hypoallergenic	−0.070 (−0.088)	0.116 (0.108)	−0.251 ** (−0.269 **)	0.096 (0.112)	0.226 ** (0.221 **)
For a special category of animal (e.g., for kittens, for neutered animals, for weight control)	−0.122 (−0.145 *)	−0.162 * (−0.197 **)	0.291 ** (0.314 **)	0.092 (0.117)	−0.188 ** (−0.214 **)

Note: The values represent Pearson’s correlation coefficients (r), while Spearman’s non-parametric rank correlation coefficients (rho) are provided in parentheses. * *p* < 0.05, ** *p* < 0.01.

**Table 9:** Correlation of the degree of trust in information sources with the advertisements that attract the greatest interest.

**Dogs** **Degree of Trust in Information Sources/Advertisements with the Greatest Interest**	**Video**	**Photo**	**Post with Pets**	**Influencers**	**Content Creators**
Veterinary	0.149 * (0.173 **)	0.088 (0.078)	0.178 ** (0.179 **)	0.066 (0.033)	0.074 (0.038)
Advertising	0.233 ** (0.223 **^)^	0.314 ** (0.310 **)	0.293 ** (0.291 **)	0.292 ** (0.325 **)	0.319 ** (0.335 **)
Influencers	0.182 ** (0.185 **)	0.241 ** (0.249 **)	0.206 ** (0.189 **)	0.548 ** (0.574 **)	0.457 ** (0.468 **)
Pet shop	0.253 ** (0.234 **)	0.341 ** (0.331 **)	0.294 ** (0.277 **)	0.122 (0.118)	0.182 ** (0.171^*^)
Social	0.129 (0.110)	0.245 ** (0.254 **)	0.217 ** (0.213 **)	0.344 ** (0.366 **)	0.397 ** (0.400 **)
**Cats Degree of Trust in Information Sources/Advertisements with the Greatest Interest**	**Video**	**Photo**	**Post with Pets**	**Influencers**	**Content Creators**
Veterinary	0.085 (0.073)	−0.197 ** (−0.215 **)	0.541 ** (0.542 **)	0.112 (0.128 *)	−0.417 ** (−0.416 **)
Advertising	0.231 ** (0.221 **)	0.186 ** (0.179 **)	0.502 ** (0.539 **)	0.408 ** (0.439 **)	0.070 (0.047)
Influencers	0.197 ** (0.203 **)	0.434 ** (0.451 **)	−0.321 ** (−0.312 **)	0.298 ** (0.305 **)	0.648 ** (0.662 **)
Pet shop	0.121 (0.107)	0.037 (0.009)	0.550 ** (0.545 **)	0.272 ** (0.303 **^)^	−0.160 * (−0.166 **)
Social	0.215 ** (0.191 *)	0.341 ** (0.351 **)	0.414 ** (0.422 **)	0.552 ** (0.541 **)	0.465 ** (0.478 **)

Note: The values represent Pearson’s correlation coefficients (r), while Spearman’s non-parametric rank correlation coefficients (rho) are provided in parentheses. * *p* < 0.05, ** *p* < 0.01.

**Table 10:** Differences between income groups in terms of the degree of interest in each type of advertising and the type of pet (dogs vs. cats).

**Dogs**	**Monthly Net Income (€):**							
	**Over 2500**		**1001–2500**		**501–1000**		**Up to 500**	
	**M**	**SD**	**M**	**SD**	**M**	**SD**	**M**	**SD**
Video	3.1	1.5	3.3	1.2	3.3	1.1	3.6	1.0
Photo	2.6	1.5	2.8	1.0	2.7	1.0	2.9	1.2
Post with pets	3.0	1.6	3.1	1.1	3.2	1.0	3.3	1.3
Influencers	1.8	1.4	1.7	1.0	1.8	.9	2.1	1.2
Content creators	1.6	0.9	2.0	1.1	2.1	1.0	2.1	1.1
**Cats**	**Monthly Net Income (€):**							
	**Over 2500**		**1001–2500**		**501–1000**		**Up to 500**	
	**M**	**SD**	**M**	**SD**	**M**	**SD**	**M**	**SD**
Video	3.3	1.3	3.1	1.2	3.0	1.1	3.1	1.1
Photo	3.5	1.3	3.2	1.2	2.7	1.0	2.9	1.1
Post with pets	3.3	1.8	2.2	1.3	2.6	1.3	2.8	1.3
Influencers	1.4	0.7	1.7	0.9	1.9	0.9	1.7	0.9
Content creators	2.5	1.9	2.9	1.2	2.3	1.1	2.0	1.2

## Data Availability

Most of the data presented in this study are contained within the text. The remaining data are available on request from the corresponding author.

## Supplementary Materials

The following supporting information can be downloaded at: https://www.mdpi.com/article/10.3390/ani16213462/s1, File S1: Full Research Questionnaire.
