# Supplementary material for: Analysis of Consumer Purchasing Behavior in Pet Food Industry: Marketing Mix Strategies Across Dog and Cat Owners
Source: Animals (Basel). 2026 Nov 2;16(21):3462. doi: 10.3390/ani16213462 (PMC13761492; doi:10.3390/ani16213462)
Supplement: Supplementary file 1 [file animals-16-03462-s001.zip › animals-4610628-supplementary.pdf]

Supplementary File S1: Full Research Questionnaire

Project Title: Investigating Pet Food Purchasing Behavior through the Marketing Mix Framework

Methodological Notes: The survey was conducted via two dedicated digital links, tailored for cat owners (31 distinct question blocks) and dog owners (32 distinct question blocks), respectively. The two instruments are identical in terms of structure, wording, and response scales, with one exception: the questionnaire for dog owners includes an additional, species-specific item in Question 6, which assesses physical confinement ("My dogs spend most of their time during the day: Free / Confined [chained, in a confinement crate]"). Consequently, Questions 6 through 31 in the cat-owner version presented below correspond to Questions 7 through 32 in the dog-owner version. For the sake of consistent alignment, all question numbers cited in the main manuscript and in Table 1 follow the standardized 31-question numbering scheme of the cat-owner instrument provided below. All questions marked with an asterisk (*) were set as mandatory fields within the digital survey interface, while multi-item matrix blocks were used to capture distinct behavioral sub-items that were analyzed independently in SPSS.

* Indicates required question

1. To continue, please select the following:*

Mark only one oval.

- I have read and understood the above information and wish to participate in the research.

GENERAL PET DATA

2. Please complete the number of your cats:*

Mark only one oval.

- 1
- 2
- 3
- >3

3. What is the breed of the cat(s)?*

Mark only one oval.

- Purebred
- Crossbred (mixed breed)
- Both (for > 1 cats)

4. How old is your cat(s)?*

- [Open-ended text field]

5. Are your cats sterilized?*

Mark only one oval.

- Yes
- No
- I do not know
- Other: ______________

[Note: In the questionnaire for dog owners, an additional question has been inserted here as Question 6: “My dogs spend the majority of their day: ( ) Free / ( ) Confined (on a chain, in a confinement crate),” shifting the numbering of the subsequent questions by +1, up to and including Question 32.]

6. My cats spend most of their time during the day:*

Mark only one oval.

- Indoors
- On the balcony
- In a garden / yard

7. In case of your absence, where do they stay and who takes care of your cats?*

Mark only one oval.

- They remain at home and a familiar person takes care of them
- At a familiar person's house
- Pet hotel or pet sitter
- Other: ______________

CAT NUTRITION

8. What type of food do you mainly provide to your cat?*

Mark only one oval.

- Commercial ready-made food
- Leftovers of your own food
- You prepare special homemade food intended only for your cat
- BARF
- Other: ______________

9. How many times a day do you feed your cat?*

Mark only one oval.

- 1 time
- 2 times
- 3 or more
- Other: _______________

10. How often do you give treats or other dietary supplements to your cat daily?*

Mark only one oval.

- I do not give treats
- 1 time / day
- 2 times / day
- 3 or more / day

11. When selecting the main food for your cat, rate how much you are influenced by the following choices.*

Mark only one oval per row. (5-point scale: Never, Rarely, Sometimes, Often, Always)

Attributes	Never	Rarely	Sometimes	Often	Always
Veterinarian recommendation	( )	( )	( )	( )	( )
Advertisement	( )	( )	( )	( )	( )
Recommendation by an acquaintance	( )	( )	( )	( )	( )
My own research	( )	( )	( )	( )	( )
Cat's preferences	( )	( )	( )	( )	( )
Animal scientist recommendation	( )	( )	( )	( )	( )

12. When selecting the main food for your cat, do you check if it is:*

Mark only one oval per row. (5-point scale: Never, Rarely, Sometimes, Often, Always)

Attributes	Never	Rarely	Sometimes	Often	Always
Organic	( )	( )	( )	( )	( )
Vegan / plant-based	( )	( )	( )	( )	( )
Cruelty free	( )	( )	( )	( )	( )
Sustainably produced	( )	( )	( )	( )	( )
Ecologically packaged	( )	( )	( )	( )	( )

13. Do you believe that nutrition directly influences the health of your cat?*

Mark only one oval.

- Yes
- No
- Other: _______________

PURCHASING BEHAVIOR

14. How much money do you spend on the nutrition of one of your cats monthly?*

Mark only one oval.

- Up to €50
- €51-100
- > €100

15. Which characteristics on a label would prompt you to purchase a product?*

Mark only one oval per row. (5-point scale: Never, Rarely, Sometimes, Often, Always)

Label Characteristics	Never	Rarely	Sometimes	Often	Always
Without preservatives	( )	( )	( )	( )	( )
High protein	( )	( )	( )	( )	( )
Low fat	( )	( )	( )	( )	( )
With natural preservatives	( )	( )	( )	( )	( )
With selected premium ingredients	( )	( )	( )	( )	( )
Grain-free	( )	( )	( )	( )	( )
Gluten-free	( )	( )	( )	( )	( )
Without additives / preservatives	( )	( )	( )	( )	( )
Hypoallergenic	( )	( )	( )	( )	( )
For a specific animal category (e.g., for kittens, for sterilized cats, for weight control)	( )	( )	( )	( )	( )

16. From where do you procure pet food?*

Mark only one oval per row. (5-point scale: Never, Rarely, Sometimes, Often, Always)

Procurement Channels	Never	Rarely	Sometimes	Often	Always
Pet shop	( )	( )	( )	( )	( )

Supermarket	( )	( )	( )	( )	( )
Veterinarian	( )	( )	( )	( )	( )
Online	( )	( )	( )	( )	( )

17. From where do you mainly get informed about your cat's nutrition?*

Mark only one oval per row. (5-point scale: Never, Rarely, Sometimes, Often, Always)

Information Sources	Never	Rarely	Sometimes	Often	Always
Veterinarian	( )	( )	( )	( )	( )
Animal scientist	( )	( )	( )	( )	( )
Internet	( )	( )	( )	( )	( )
Social Media	( )	( )	( )	( )	( )
Articles	( )	( )	( )	( )	( )
Trainer	( )	( )	( )	( )	( )
Other cat owners	( )	( )	( )	( )	( )

18. When selecting the main food for your cat, how often are you influenced by the following criteria?*

Mark only one oval per row. (5-point scale: Never, Rarely, Sometimes, Often, Always)

Criteria	Never	Rarely	Sometimes	Often	Always
Price	( )	( )	( )	( )	( )
Ingredients	( )	( )	( )	( )	( )
Nutritional value	( )	( )	( )	( )	( )
Brand	( )	( )	( )	( )	( )
Veterinarian recommendation	( )	( )	( )	( )	( )
Advertising	( )	( )	( )	( )	( )
Packaging (e.g., photo, ecological, practical)	( )	( )	( )	( )	( )

INFLUENCE OF ADVERTISEMENTS

19. How often have you seen an advertisement for cat food or dietary supplements in the last month?*

Mark only one oval per row. (5-point scale: Never, Rarely, Sometimes, Often, Always)

Advertising Medium	Never	Rarely	Sometimes	Often	Always
Television	( )	( )	( )	( )	( )
Internet	( )	( )	( )	( )	( )
Social	( )	( )	( )	( )	( )
Pet shops	( )	( )	( )	( )	( )
Print medium (e.g., magazine)	( )	( )	( )	( )	( )

20. Regarding advertisements for cat nutrition products, select to what extent the following apply.*

Mark only one oval per row. (5-point scale: Not at all, A little, Moderately, A lot, Very much)

Statements	Not at all	A little	Moderately	A lot	Very much
They present honest and reliable information about the products.	( )	( )	( )	( )	( )
They influence me when choosing a product.	( )	( )	( )	( )	( )
They should be controlled/regulated more.	( )	( )	( )	( )	( )
They are misleading.	( )	( )	( )	( )	( )
They try to influence me emotionally.	( )	( )	( )	( )	( )

21. How much do you trust each of the following sources for your cat's nutrition?*

Mark only one oval per row. (5-point scale: Not at all, A little, Moderately, A lot, Very much)

Sources	Not at all	A little	Moderately	A lot	Very much
Veterinarian	( )	( )	( )	( )	( )
Advertisement	( )	( )	( )	( )	( )
Influencers	( )	( )	( )	( )	( )
Pet shop	( )	( )	( )	( )	( )
Social	( )	( )	( )	( )	( )

22. Do you believe that companies use misleading terms or exaggerated promises? If yes, which terms seem unclear to you?*

- [Open-ended qualitative text field]

23. Which type of advertisement do you find most interesting:*

Mark only one oval per row. (5-point scale: Not at all, A little, Moderately, A lot, Very much)

Type of Advertisement	Not at all	A little	Moderately	A lot	Very much
Video	( )	( )	( )	( )	( )
Photo	( )	( )	( )	( )	( )
Post with pets	( )	( )	( )	( )	( )
Influencers	( )	( )	( )	( )	( )
Content creators	( )	( )	( )	( )	( )

24. What attracts your attention the most in a pet food advertisement?*

Mark only one oval per row. (5-point scale: Not at all, A little, Moderately, A lot, Very much)

Attention Drivers	Not at all	A little	Moderately	A lot	Very much
Image of the animal (e.g., shiny coat, energy)	( )	( )	( )	( )	( )
Packaging	( )	( )	( )	( )	( )
Price	( )	( )	( )	( )	( )
Nutritional claims	( )	( )	( )	( )	( )
Logo	( )	( )	( )	( )	( )
Aesthetics	( )	( )	( )	( )	( )
Presence of an "expert"	( )	( )	( )	( )	( )
Branding (Brand)	( )	( )	( )	( )	( )

25. Regarding the brand name (Brand) of a cat nutritional product, select how much the following apply.*

Mark only one oval per row. (5-point scale: Not at all, A little, Moderately, A lot, Very much)

Brand Perceptions	Not at all	A little	Moderately	A lot	Very much
The history/recognition of the brand influences me positively in the choice.	( )	( )	( )	( )	( )
I remain loyal to a specific company.	( )	( )	( )	( )	( )
The charitable action of the company influences me positively.	( )	( )	( )	( )	( )

I am interested in knowing the production method of its products.	( )	( )	( )	( )	( )
I am interested in knowing its employees.	( )	( )	( )	( )	( )
It is important for it to present scientifically substantiated data.	( )	( )	( )	( )	( )

26. Would you like companies to conduct surveys/questionnaires more frequently asking for the consumer's opinion?*

Mark only one oval.

- Yes
- No
- Other: _______________

GENERAL OWNER DATA

27. How long have you been a cat owner?*

Mark only one oval.

- 1 - 5 years
- 5 - 10 years
- > 10 years

28. What is your gender?*

Mark only one oval.

- Man
- Woman
- Other: _______________

29. Your age is:*

Mark only one oval.

- up to 24
- 25-44
- 45-64
- 65 years and over

30. Your monthly net income (€) is:*

Mark only one oval.

- up to 500

- 501-1000
- 1001-2500
- over 2500
- I do not wish to answer

31. If you want to share something more or ideas for improvement, you can do it here.*

- [Open-ended qualitative text field]
